# Iron(III)-salophene catalyzes redox cycles that induce phospholipid peroxidation and deplete cancer cells of ferroptosis-protecting cofactors

**DOI:** 10.1016/j.redox.2024.103257

**Published:** 2024-06-26

**Authors:** Fengting Su, Hubert Descher, Minh Bui-Hoang, Hermann Stuppner, Ira Skvortsova, Ehsan Bonyadi Rad, Claudia Ascher, Alexander Weiss, Zhigang Rao, Stephan Hohloch, Solveigh C. Koeberle, Ronald Gust, Andreas Koeberle

**Affiliations:** a Michael Popp Institute, Center for Molecular Biosciences Innsbruck (CMBI), University of Innsbruck, Innsbruck, Austria; b Institute of Pharmacy/Pharmaceutical Chemistry, Center for Molecular Biosciences Innsbruck (CMBI), University of Innsbruck, Innsbruck, Austria; c Unit of Pharmacognosy, Institute of Pharmacy, Center for Molecular Biosciences Innsbruck (CMBI), University of Innsbruck, Innsbruck, Austria; d EXTRO-Lab, Department of Therapeutic Radiology and Oncology, Medical University of Innsbruck, Innsbruck, Austria; e Institute for Biomedical Aging Research, University of Innsbruck, Innsbruck, Austria; f Institute for General, Inorganic and Theoretical Chemistry, University of Innsbruck, Innsbruck, Austria

**Keywords:** Ferroptosis, Iron complexes, Reactive oxygen species, Redox mechanism, Lipidomics, NADPH

## Abstract

Ferroptosis, a lipid peroxidation-driven cell death program kept in check by glutathione peroxidase 4 and endogenous redox cycles, promises access to novel strategies for treating therapy-resistant cancers. Chlorido [N,N′-disalicylidene-1,2-phenylenediamine]iron (III) complexes (SCs) have potent anti-cancer properties by inducing ferroptosis, apoptosis, or necroptosis through still poorly understood molecular mechanisms. Here, we show that SCs preferentially induce ferroptosis over other cell death programs in triple-negative breast cancer cells (LC_50_ ≥ 0.07 μM) and are particularly effective against cell lines with acquired invasiveness, chemo- or radioresistance. Redox lipidomics reveals that initiation of cell death is associated with extensive (hydroper)oxidation of arachidonic acid and adrenic acid in membrane phospholipids, specifically phosphatidylethanolamines and phosphatidylinositols, with SCs outperforming established ferroptosis inducers. Mechanistically, SCs effectively catalyze one-electron transfer reactions, likely via a redox cycle involving the reduction of Fe(III) to Fe(II) species and reversible formation of oxo-bridged dimeric complexes, as supported by cyclic voltammetry. As a result, SCs can use hydrogen peroxide to generate organic radicals but not hydroxyl radicals and oxidize membrane phospholipids and (membrane-)protective factors such as NADPH, which is depleted from cells. We conclude that SCs catalyze specific redox reactions that drive membrane peroxidation while interfering with the ability of cells, including therapy-resistant cancer cells, to detoxify phospholipid hydroperoxides.

## 1 Introduction

Induction of cell death by ferroptosis is an emerging anti-cancer strategy with promising pre-clinical outcomes in aggressive, therapy-resistant tumors, some of which even gain ferroptosis susceptibility [[1], [2], [3], [4]]. Ferroptosis is distinct from other cell death programs, including apoptosis, necroptosis, pyroptosis, and autophagy, and is driven by excessive membrane peroxidation [5,6]. The latter is dependent on either the labile Fe(II) pool, which generates hydroxyl (OH) radicals from hydrogen peroxide (H_2_O_2_) via the Fenton reaction [7,8], or iron incorporated into enzymes that produce or contribute to the production of phospholipid hydroperoxides, such as lipoxygenases (LOX) [9,10] and cytochrome P450 oxidoreductase (POR) [11,12]. Particularly sensitive to peroxidation are the allylic positions of polyunsaturated fatty acids (PUFAs) [13], with the levels of arachidonic acid (20:4) and adrenic acid (22:4) in phosphatidylethanolamines (PEs) and, less studied, phosphatidylinositols (PIs) being strongly associated with ferroptosis susceptibility [14,15]. Tightly regulated repair and protection systems keep ferroptosis in check [1,16,17]: GPX4 critically counteracts ferroptosis by reducing phospholipid hydroperoxides to non-toxic alcohols using glutathione (GSH) as a co-substrate [7,18]. Other protective redox cycles are based on ferroptosis suppressor protein 1 (FSP1)/coenzyme (Co)Q10 and GTP cyclohydrolase 1 (GCH1)/tetrahydrobiopterin (BH_4_) [[19], [20], [21], [22], [23]]. The former uses nicotinamide adenine dinucleotide phosphate (NAD(P)H) to regenerate CoQ10 beyond the inner mitochondrial membrane [20,21].

Various strategies to induce ferroptosis are being explored [1,2,24,25] to eliminate specific cancer cell populations, such as cisplatin-resistant non-small cell lung cancer, 5-fluorouracil-resistant colorectal carcinoma cells, and apoptosis-resistant pancreatic ductal adenocarcinoma cells to name a few [2,26]. While several clinically used drugs have subsequently been found to induce ferroptosis, among other activities [27], no rationally designed small molecules have yet entered clinical trials. Typically, the most stable iron-salene and iron-salophene complexes (SCs) consist of a central Fe(III) ion coordinatively bound to N,N′-bis(salicylidene)ethylenediamine or *N*,*N*′-bis(salicylidene)-1,2-phenylenediamine and an interchangeable axial ligand. Together with their dimeric μ-oxo complexes, they are potent inducers of ferroptosis in certain cancer cell lines [[28], [29], [30]], including leukemia cells [28]. However, depending on the system and experimental settings, they can also induce other cell death programs, such as apoptosis [[31], [32], [33], [34], [35], [36], [37], [38], [39], [40], [41]] and necroptosis [28,34] or interfere with cell cycle progression [31,38]. This multimodal cell death induction might explain the ability of SC to effectively kill (chemo-resistant) cancer cells in culture, in grafted mice, and in rodent cancer models [31,33,36,38,39,42], while being less toxic to non-malignant cells [31,35,36,43] – a feature shared by several other ferroptosis inducers [2]. Accordingly, severe hepatic and renal side effects are only seen at high dosages in rodents [31,39]. Since SCs are magnetically active, they can be used to induce hyperthermia by alternating magnetic field (AMF) [37,[43], [44], [45]], allowing combination therapies.

A large number of SC derivatives have been synthesized, with the substituents defining the cytotoxic potency and directing the cell death program [28,36,41]. Some SCs can mimic the catalytic activity of distinct iron-containing oxidoreductases, such as cytochrome P450 monooxygenases [46], dioxygenases [47], superoxide dismutase [48] and amino acid oxidases [49], partially under the formation of high-valent oxoiron species. Mechanistically, SCs induce chromatin fragmentation [31,32,38,48,50,51], activate the caspase cascade and thereby trigger apoptosis via the mitochondrial pathway [31,32,38,39], interfere with mitogenic signaling and cause cell cycle arrest in G1-or *S*-phase [31,38], induce oxidative stress [34,38] and activate stress-activated protein kinases [38]. Alternatively, SCs increase mitochondrial reactive oxygen species (ROS) levels and induce ferroptosis, though the molecular mechanism remained obscure. Compensation studies with antioxidants suggest that both ferroptosis and apoptosis induction by SCs are dependent on ROS [28]. Note that SCs do not release Fe(III) ions (under non-acidic conditions), even in the presence of the iron chelator deferoxamine (DFO) [28], which suppresses the cytotoxic activity of SCs, as expected for a ferroptosis inhibitor [52].

Here, we investigated the anti-tumoral mechanism of SCs with a focus on their ferroptosis-triggering component. We identified human triple-negative MDA-MB-231 breast cancer cells as particularly sensitive to ferroptosis induction by SCs and demonstrate the efficacy of SCs against chemoresistant, radioresistant, and invasive breast cancer and osteosarcoma cell lines. Exposure of cells to lipophilic (but not hydrophilic) SCs cause massive phospholipid peroxidation, specifically of arachidonic acid (20:4) and adrenic acid (22:4) in phosphatidylethanolamines (PE) and phosphatidylinositols (PI), via multiple mechanisms. First, SCs act as redox catalysts that reversibly form oxo-bridged dimeric complexes and undergo one-electron redox cycling thereby propagating membrane peroxidation even in the absence of a cellular environment or hydrogen peroxide. Second, the complexes decompose H_2_O_2_ by generating ROS, i.e., organic solvent radicals, which might produce initial phospholipid hydroperoxides, whereas OH radicals do not seem to be formed, contrary to our expectations. Third, SCs catalyze the two-electron oxidation of specific redox-sensitive molecules by H_2_O_2_, including viable redox cofactors such as NADPH, whose dysregulation is closely linked to oxidative stress and cell death by apoptosis and ferroptosis. This multifaceted interference with redox homeostasis may explain why SCs are more effective in inducing membrane peroxidation than mobile Fe(II) or GPX4 inhibitors and provide insights into how they may engage different cell death programs.

## 2 Materials and methods

### 2.1 Materials

The SCs **1**, **2**, and **3** (purity ≥95 %) and the μ-oxo derivatives μ-oxo-**1**, μ-oxo-**2** and μ-oxo-**3** (purity ≥95 %) were synthesized following previously published procedures [28,29], dissolved in DMSO (10 mM) or methanol (5 mM for EPR), and aliquots were stored at −20 °C under argon. The hydrophilic sulfonates **4**, **5**, **6**, and **7** (purity ≥95 %) were synthesized as recently described [29,53] and dissolved in H_2_O (2 mM) or DMSO (2 mM), and aliquots were stored at −20 °C under argon. Phospholipids and lipid standards were obtained from Merck (Darmstadt, Germany), dissolved in chloroform, aliquoted, and stored protected from light at −80 °C under argon. RSL3, erastin, ferrostatin-1 (Fer-1), necrostatin-1 (Nec-1), MCC950.Na, 3-methyladenine (3-MA), necrostatin-2 (Nec-2), wortmannin, CAY10698, nordihydroguaiaretic acid (NDGA), and BLX3887 were purchased from Cayman (Vienna, Austria). FeCl_3_, CJ-13610, *N*-acetyl-*l*-cysteine (NAC), ciclopirox, Q-VD-OPh, baicalein, H_2_O_2_, staurosporine, 5,5′,6,6′-tetrachloro-1,1′,3,3′tetraethyl-benzimidazolylcarbocyanine iodide (JC-1), carbonyl cyanide-*p*-trifluoromethoxyphenylhydrazone (FCCP) solution (#SML2959), and oligomycin were obtained from Sigma Aldrich (Vienna, Austria). Liproxstatin-1 (Lip-1), and β-mercaptoethanol (β-ME) were bought from Fisher Scientific (Milan, Italy), and NADPH and FeSO_4_ were ordered from Carl Roth (Karlsruhe, Germany).

### 2.2 Cell culture

Cell lines were obtained from the American Type Culture Collection (ATCC, Manassas, VA) unless otherwise stated, routinely maintained at 37 °C in a 5 % CO_2_ atmosphere, and passaged after trypsinization (trypsin-EDTA, Merck, #59418C) every 3–4 days before reaching confluence. Human mammary epithelial MCF12A cells (passage number 2–10) were grown in DMEM/F12 (Gibco, Darmstadt, Germany; #11554546), 100 ng/mL cholera toxin (Merck, #C8052), 0.01 mg/mL bovine insulin (Sigma Aldrich, #I5500), 20 ng/mL human epidermal growth factor (Sigma Aldrich, E9644), 500 ng/mL hydrocortisone. Human triple-negative MDA-MB-231 breast cancer cells (passage number 46–85), human osteosarcoma U2OS (passage number 35–43) and MG63 cells (passage number 38–46), and human HepG2 heptocarcinoma cells (passage number 19–23) were cultured in DMEM (glucose 4.5 g/L) medium (Gibco, #11965092), human MCF7 breast cancer cells (passage number 6–18) were cultured in DMEM (glucose 4.5 g/L) with 1 mM sodium pyruvate (Gibco, #12539059) and 10 μg/mL human recombinant insulin (Sigma Aldrich, #I9278), and human A549 lung carcinoma cells (Sigma Aldrich, #86012804; passage number 13–17) were cultured in RPMI-1640 medium supplemented with *l*-glutamine (Merck, #R8758), and human T-47D luminal A breast cancer cells were cultured in RPMI-1640 medium supplemented with *l*-glutamine (Merck, #R8758) and 10 μg/mL bovine insulin. Radio-resistant T-47D cells (T-47D_RR) were generated from parental T-47D cells by repeated exposure to ionizing radiation (10 Gy) (16 MV x-ray) [54]. Invasive T-47D cells (T-47D_Invasive) were generated from parental T-47D cells by repeated selection of cells that efficiently migrate in Boyden chambers through uncoated 8 μm pore membranes towards 10 % fetal calf serum (FCS) as attractant [55]. All culture media were supplemented with 10 % FCS (Gibco, #2453039), 100 U/mL penicillin and 100 g/mL streptomycin (Sigma Aldrich, #P0781). Cells were regularly tested for mycoplasma contamination (MycoAlert™ PLUS Mycoplasma Detection Kit, Lonza, Basel, Switzerland) and their morphology was inspected. MDA-MB-231, MCF7, U2OS, MG63, HepG2, and T-47D cells were authenticated by Multiplexion (Friedrichshafen, Germany) in December of 2020, April of 2022, and June of 2022, respectively, using single nucleotide polymorphism (SNP) profiling (Multiplex Cell Line Authentication, https://www.multiplexion.de/en/cell-line-testing-service/multiplex-human-cell-line-authentication).

### 2.3 Establishment of doxorubicin-resistant U2OS cells

Doxorubicin-resistant U2OS cells (U2OS_Dox) were generated by exposing cells (at 80 % confluence) to doxorubicin (Szabo Scandic, #15007; 3.75 nM) and increasing the concentration stepwise every two weeks until the concentration reached 500 nM after four months. Resistant cells were maintained in medium containing 500 nM doxorubicin, which was replenished every two days. To study the effect of SCs on cell viability, doxorubicin-resistant U2OS cells were cultured in doxorubicin-free medium for two days before starting the experiments.

### 2.4 Cell count and morphology

Viable cell number and membrane intactness were determined by trypan blue staining using a Vi-CELL Series Cell Counter (Beckman Coulter, Krefeld, Germany). Cell morphology was visualized using an AE31E light microscope (Motic, Hong Kong, China) equipped with a Moticam 10+ camera (Motic). To monitor cell proliferation over 72 h, 5 × 10^3^ to 2.5 × 10^4^ cells were seeded in a 96-well plate (Fisher Scientific, #15533115) for 24 h, 48 h, and 72 h.

### 2.5 Assessment of cell viability based on dehydrogenase activity

Cell viability was estimated from the conversion of 3-(4,5-dimethyldiazol-2-yl)-2,5-diphenyltetrazolium bromide (MTT) (Cayman, #37009) by cellular dehydrogenases. Briefly, cells were seeded in 96-well plates and, after 24 h at 37 °C and 5 % CO_2_, treated with vehicle (DMSO, 0.5 %) or test compounds for 48 h in 100 μL of culture medium. Ethanol (Fisher Scientific, #10048291; 16.7 %) and staurosporine (Sigma Aldrich, #S4400; 1 μM) were used as controls. To each well, 20 μL MTT (5 mg/mL in phosphate-buffered saline (PBS) pH 7.4, sterile filtered) was added and the incubation continued for 1.5 h. Cells were lysed and MTT was solubilized by adding 100 μL SDS buffer (Carl Roth, #2326.2; 10 % SDS in 20 mM HCl, pH 4.5) with shaking in the dark for 20 h. Absorbance was measured at 570 nm using a SpectraMAX iD3 spectrometer (Molecular Devices, San José, CA) and normalized to vehicle (100 % cell viability) and ethanol control (0 % cell viability).

### 2.6 Crystal violet staining of breast cell colonies

MCF-12A cells (1 × 10^4^) and MDA-MB-231 cells (1 × 10^4^) were seeded in 6-well plates (Fisher Scientific, #10380291) in 2 mL of culture medium for 24 h, then the medium was replaced every 2 days with fresh culture medium containing vehicle (0.1 % DMSO) or test compounds. After 10 days, the cells were subjected to crystal violet staining. Adherent breast cancer cells were washed twice with PBS pH 7.4, fixed with methanol (−20 °C) for 10 min at room temperature, and stained with a solution of 0.5 % (m/v) crystal violet (Fisher Scientific, #11435027) in methanol/H_2_O = 20/80 (v/v) for another 10 min. After washing with H_2_O, cells were imaged using an AE31E light microscope (Motic) equipped with a Moticam 10+ camera (Motic). Cell numbers in microscopic images were determined by automated cell tracking using ImageJ (1.53k, National Institute of Health, Bethesda, Maryland).

### 2.7 Determination of cellular ROS levels

Cellular ROS were determined using the Cellular ROS Assay Kit (Red) (Abcam, Cambridge, UK; #ab186027) according to the manufacturer's instructions. Briefly, MDA-MB-231 cells (2 × 10^4^) were seeded in 96-well plates (Fisher Scientific, #10281092) with 100 μL of medium and, after 24 h, incubated with 100 μL of ROS Red working solution (containing 2 μL/mL ROS Red stock solution in assay buffer) for 1 h at 37 °C and 5 % CO_2_. Cells were then treated with vehicle (DMSO, 0.5 %), test compounds (1 μM), or H_2_O_2_ (1 mM) for 2 h at 37 °C and 5 % CO_2_ before fluorescence (Ex/Em = 520/605 nm) was measured using a SpectraMAX iD3 spectrometer (Molecular Devices) in bottom read mode.

### 2.8 Detection of cellular lipid hydroperoxides

MDA-MB-231 cells (5 × 10^5^) in 6-well plates were incubated at 37 °C in 5 % CO_2_ for 24 h, washed with serum-free DMEM, and treated with vehicle (DMSO, 0.5 %) or test compounds (1 μM) in the absence or in the presence of Fer-1 (3 μM) in DMEM at 37 °C in 5 % CO_2_ for 6 h. After two washes with 2 mL Hanks’ balanced salt solution (HBBS) (Fisher Scientific, #11570476), cells were further incubated with Liperfluo (Gerbu Biotechnik GmbH, Heidelberg, Germany; #L248; 10 μM) in HBSS for 30 min. Cells were harvested by trypsinization and washed twice with HBSS, then resuspended in HBSS at 5 × 10^5^ cells/mL, and analyzed with a Guava easyCyte 8HT flow cytometer (Merck Millipore, Vienna, Austria) at λ_Ex/Em_ = 488/550 nm. The histograms in Fig. 2D were generated using FlowJo 7.6.5 (Becton Dickinson, Ashland, USA). The gating strategy is outlined in Supplementary Fig. 1A.

### 2.9 Sample preparation for quantitative (redox) lipidomics

Phospholipids were extracted from cell pellets by sequential addition of PBS pH 7.4, methanol, chloroform, and saline (final ratio: 14:34:35:17) [56,57]. The chloroform layer was dried using an Eppendorf Concentrator Plus System (Eppendorf, Hamburg, Germany) and stored at −80 °C under argon. Internal standards: 1,2-dimyristoyl-*sn*-glycero-3-phosphatidylcholine (DMPC) and 1,2-dimyristoyl-*sn*-glycero-3-phosphatidylethanolamine (DMPE).

### 2.10 Quantitative analysis of oxidized and non-oxidized phospholipids

PC, PE and PI were diluted in methanol and separated at a flow rate of 0.75 mL/min at 45 °C on an ACQUITY UPLC® BEH C8 column (2.1 × 100 mm, 130 Å, 1.7 μm; Waters, Milford, MA) with an ExionLC AD UHPLC system (Sciex, Framingham, MA) [58]. The gradient of mobile phase A (acetonitrile/water, 95/5, 2 mM ammonium acetate) and mobile phase B (water/acetonitrile, 90/10, 2 mM ammonium acetate) was increased linearly from A/B = 75/25 to 85/15 within 5 min and then to 100 % A within 2 min followed by isocratic elution at 100 % A for another 2 min. The UHPLC system was coupled to a QTRAP 6500^+^ mass spectrometer (Sciex) equipped with an IonDrive Turbo V ion source and a TurboIonSpray probe for electrospray ionization. The mass spectrometric source and compound parameters for the analysis of non-oxidized and oxidized phospholipids are listed in Table S1. Mass spectra were acquired using Analyst 1.7.1 (QTRAP6500^+^, Sciex) and processed using Analyst 1.6.3 (Sciex) [59].

Non-oxidized glycerophospholipids were detected after fragmentation to both fatty acid anions by multiple reaction monitoring (MRM) in the negative ionization mode, with quantitation being based on the mean of both transitions [60]. Absolute amounts of PC, PE and PI were calculated by normalizing the signals to cell number and a subgroup-specific deuterated internal standard. Relative intensities (indicating the proportion of lipids) were obtained by summing all signals analyzed within the subgroup (e.g., PE) and expressing the individual signals of lipid species or lipid subfractions as a percentage of this sum (=100 %). Saturated fatty acid (SFA), MUFA, and PUFA (≥2 double bonds) fractions in phospholipids were calculated from the mean signal intensities of the transitions to *sn*-1 and *sn*-2 fatty acid anions, which were equally assigned (50 %, each) to the corresponding subfractions.

Oxidized phospholipid anions (PE and PI: [*M* − H]^-^; PC: [M + OAc]^-^) were detected after fragmentation to both fatty acid anions, i.e., the SFA or MUFA anion and the oxidized PUFA (20:4 and 22:4) anion, which has one, two, or three oxygens incorporated and is optionally further fragmented (Table S2). Signals were considered only if their retention times were consistent with changes expected from their acyl carbon and double bond numbers and within predefined ranges (Table S3, **S4**, and **S5**). Retention time windows for oxidized PC and oxidized PE were predicted based on the analysis of oxPAPC (Avanti Polar Lipids, Alabaster, AL; 1[O]: 2.8–4.6 min; 2[O]: 2.9–4.3 min; 3[O]: 1.45–2.3 min) and PE(16:0_20:4) (Avanti Polar Lipids), the latter enzymatically oxygenated with soybean lipoxidase (type V) (Merck, #L6632) (1[O]: 2.79–2.96 min; 2[O]: 2.84–3.04 min) [61]. These experimentally defined retention time windows have been extended to include potential regioisomers [62] and adapted to additional PC, PE, and PI species based on the effective carbon/double bond number model, as listed in Table S3, **S4**, and **S5**. Quantitation is based on the most intense signals of oxidized fatty acid anion fragments. The fractions of phospholipids with one 1[O], two 2[O], or three oxygens 3[O] incorporated comprise multiple isomeric species that were summed and normalized to DMPC (for oxidized PC and oxidized PI) or DMPE (for oxidized PE) and cell number.

### 2.11 Enzymatic synthesis of oxidized PE(16:0/20:4) for the use as standard

5 mg PE(16:0/20:4) (Avanti Polar Lipids, #850759C) in 3.43 mL 3 % aqueous sodium deoxycholate solution (Merck; 3.43 mL) and 24.43 mL 200 mM Tris pH 8.6 was incubated with 1 mg type V lipoxidase from Glycine max (Merck, #SLCC4512) for 20 min with stirring at room temperature. Phospholipids were recovered by solid phase extraction using a Sep-Pak C18 6 cc Vac cartridge (500 mg sorbent, Waters). The cartridges were washed with 10 mL H_2_O, and the phospholipids were eluted with 5 mL methanol and evaporated to dryness using a TurboVap LV automated solvent evaporation system (Biotage Sweden AB). The residue was dissolved in ethanol (1.6 mL) and diluted 1:100 with methanol for the measurement of oxidized PE; an aliquot was stored in ethanol under argon at −80 °C.

### 2.12 Preparation of artificial membranes from egg PC

Egg l-α-PC (Sigma Aldrich, #840051P) (75 mg) was dissolved in a minimal volume of chloroform. The solvent was evaporated under argon to leave a thin film on the wall of the vial. The lipid film was left under vacuum for 1 h to remove any residual solvent and then hydrated with 10 mM PBS pH 7.4, 150 mM NaCl (4.83 mL). The lipid suspension (20 mM) was subjected to 10 freeze-thaw-sonication cycles, each consisting of 4 min in dry ice, 4 min thawing at room temperature, and 4 min sonication (Ultrasonic cleaning baths, VWR USC1200T, HF 45 kHz 180W). The lipid suspension was then extruded 20–25 times using a LiposoFast Liposome Factory mini-extruder equipped with a 100 nm polycarbonate membrane (Sigma-Aldrich, #Z373419) to obtain liposomes (average diameter: 118.8 nm; polydispersity index: 0.228; (ZetaSizer Nano ZSP, Malvern Panalytical Ltd, UK).

### 2.13 Monitoring of phospholipid peroxidation in artificial membranes

Phospholipid peroxidation of artificial liposomal membranes was determined using a modified version of the fluorescence-enabled inhibited autoxidation (FENIX) assay [63]. Briefly, liposomes (1 mM in PBS pH 7.4) were combined in a black 96-well polypropylene plate (Greiner, #655087) with C11-BODIPY (Cayman, #27086; 1 μM) and incubated in the presence or in the absence of liproxstatin-1 (Lip-1, Fisher Scientific, #16458017; 1 μM), NADH (Fisher Scientific, #10711911; 10–100 μM), or NADPH (Carl Roth, #AE14.2; 10–100 μM) for 10 min at 37 °C, followed by vigorous mixing for 5 min. Autoxidation was initiated by the addition of test compounds (10 μM) in the presence or in the absence of H_2_O_2_ (10 μM). AAPH (Sigma Aldrich, #440914; 10 μM) was used as positive control. After mixing for 5 min, the plate was equilibrated at 37 °C for 10 min before fluorescence (λ_Ex/Em_ = 498/528 nm) was time-dependently acquired for 300 min using a SpectraMAX iD3 spectrometer (Molecular Devices) in bottom-read mode.

### 2.14 Cell-free oxidation of PE(16:0/20:4)

PE(16:0/20:4) (Avanti Polar Lipids; 83 μg) was mixed with 3 % aqueous sodium deoxycholate solution (0.65 mL) and 250 mM Tris pH 8.6 (6.1 mL) while shaking at room temperature. The mixture was incubated with vehicle (DMSO, 1 %) or **1** (10 μM) in the absence of or in the presence of H_2_O_2_ (10 μM, 0.75 mL) for 2 h at 37 °C, and oxidized phospholipids were extracted from an aliquot of 7.5 mL by solid-phase extraction [as described for the enzymatic synthesis of oxidized PE(16:0/20:4)] and analyzed by UPLC-MS/MS, using the chromatographic and mass spectrometric settings detailed above.

### 2.15 Oxidation of PE(18:0_20:4) in breast cancer cell homogenates

MDA-MB-231 cells (3.5 × 10^6^) were lysed on ice in 170 μL lysis buffer [20 mM Tris-HCl pH 7.4, 150 mM NaCl, 2 mM EDTA (Fisher Scientific, #15815288), 1 % Triton X-100 ((Fisher Scientific, #10671652)]. After sonication (3 × 5 s, on ice, 125 W, 35 % amplitude; Q125, Qsonica, Newtown, USA), the homogenate was incubated with vehicle (DMSO, 1 %), FeSO_4_ (100 μM), or **1** (1–100 μM) in the absence or in the presence of H_2_O_2_ (100 μM) at 37 °C for 2 h, and oxidized phospholipids were extracted and analyzed by UPLC-MS/MS as described for the cell-free oxidation of PE(18:0_20:4).

### 2.16 Quantitation of the labile iron pool

MDA-MB-231 cells (1 × 10^6^/25 cm^2^) incubated for 24 h were treated with vehicle (DMSO, 0.1 %), erastin, RSL3, or test compounds for an additional 24 h at 37 °C and 5 % CO_2_. Cells were collected by trypsinization, washed three times with PBS pH 7.4, and lysed in 200 μL lysis buffer for 5 min. After sonication (3 × 5 s, on ice) and centrifugation (21,000×*g*, 10 min, 4 °C), the supernatants (100 μL) were mixed with 220 μL of 2.5 M ammonium acetate buffer pH 4.5 containing 2.73 mM ferrene (Sigma Aldrich, #82940), 5.45 mM ascorbic acid (Sigma Aldrich, #A92902), and 6.54 mM thiourea (Sigma Aldrich, #T8656). Samples were incubated overnight at room temperature, centrifuged (15,000×*g*, 5 min), and the absorbance was measured at 595 nm using a SpectraMAX iD3 spectrometer (Molecular Devices). Absolute amounts of labile iron were assessed by 8-point external calibration (0–0.4 mmol FeSO_4_ in 100 μL H_2_O) and normalized to the protein concentration, which was determined in the supernatant of the cell homogenate after sonication and centrifugation using a DC protein assay kit (Bio-Rad Laboratories GmbH, Munich, Germany; #5000116).

### 2.17 Determination of Fenton activity

Test compounds were incubated with salicylic acid (Sigma Aldrich, #247588; 0.52 mM) and H_2_O_2_ (Sigma Aldrich, #H1009; 37–225 mM) in ethanol/H_2_O/DMSO = 31/69/1 (v/v/v) for 30 min at 37 °C. The OH radical-dependent hydroxylation of salicylic acid was measured at 520 nm using a SpectraMAX iD3 spectrometer (Molecular Devices). Data were corrected by subtracting the basal absorption of individual components (such as salicylic acid and **1**).

### 2.18 Protein extraction and immunoblotting

MDA-MB-231 cells (5 × 10^5^) were seeded in 6-well plates, incubated for 24 h at 37 °C in 5 % CO_2_, and treated with vehicle (DMSO, 0.1 %) or test compounds for another 24 h. Cells were harvested, washed with PBS pH 7.4, and cell pellets were resuspended in ice-cold lysis buffer supplemented with 5 mM sodium fluoride (Fisher Scientific, # 11,439,933), 10 μg/mL leupeptin (Fisher Scientific, # 10,736,392), 60 μg/mL soybean trypsin inhibitor (Sigma Aldrich, #T9128), 1 mM phenylmethanesulfonyl fluoride (Carl Roth, #6367.1), 2.5 mM sodium pyrophosphate (Fisher Scientific, #10368580), and 1 mM sodium vanadate (Carl Roth, #0735.1). After sonication (3 × 5 s, on ice, 125 W, 35 % amplitude; Q125, Qsonica) and centrifugation (12,000×*g*, 5 min, 4 °C), the protein concentration of the lysates was determined using a DC protein assay kit (Bio-Rad Laboratories GmbH). Samples were mixed with 5 × SDS/PAGE sample loading buffer [125 mM Tris-HCl pH 6.5, 25 % (m/v) sucrose, 5 % SDS (m/v), 0.25 % (m/v) bromophenol blue, and 5 % (v/v) β-mercaptoethanol] and heated at 95 °C for 5 min. Aliquots (15 μg protein) were separated on 10 % SDS-PAGE gels and transferred to Amersham Protran 0.45 μm NC nitrocellulose membranes (Carl Roth, #4675.1), which were blocked with 5 % nonfat milk for 1 h at room temperature and incubated with rabbit *anti*-GPX4 (EPNCIR144, #ab125066; 1:500; Abcam), rabbit *anti*-phospho-RIP (Ser166) (D1L3S, #65746; 1:500; Cell Signaling, Danvers, MA), rabbit anti-RIP (D94C12, #3493; 1:500; Cell Signaling), rabbit *anti*-phospho-MLKL (Ser358) (D6H3V, #91689; 1:500; Cell Signaling), rabbit *anti*-MLKL (D2I6N, #14993; 1:500; Cell Signaling), rabbit anti-cleaved caspase-3 (Asp175) (5A1E, #9664; 1:500; Cell Signaling), rabbit *anti*-caspase-3 (D3R6Y, #14220; 1:500; Cell Signaling), rabbit *anti*-PARP (46D11, #9532; 1:1000, Cell Signaling), rabbit anti-cleaved PARP (Asp214) (D64E10, #5625; 1:500, Cell Signaling), anti-catalase (#21260-1-AP, 1:1000; Proteintech, Planegg-Martinsried, Germany), anti-SOD1 (#10269-1-AP; 1:1000; Proteintech), *anti*-GPX1 (#29329-1-AP; 1:1000; Proteintech), *anti*-TXNRD1 (#11117-1-AP; 1:1000; Proteintech), and mouse *anti*-β-actin (Cell Signaling, 8H10D10, #3700; 1:1000) overnight at 4 °C. Washed membranes were incubated with the secondary antibodies DyLight® 680 goat anti-rabbit IgG (Thermo Fisher Scientific, Waltham, MA; #35569; 1:10,000) and DyLight® 800 goat anti-mouse IgG (Thermo Fisher Scientific, #SA5-10176; 1:10,000) for 1 h at room temperature. Proteins were visualized using a Fusion FX7 Edge Imaging System (spectra light capsules: C680, C780; emission filters: F-750, F-850; VILBER Lourmat, Collegien, France) [58]. Densitometric analysis was performed using Evolution-Capt Edge software version 18.06 (VILBER Lourmat), and background was subtracted based on the valley-to-valley approach. Uncropped versions of the Western blots in Fig. 4D, Supplementary Fig. 4B, and Supplementary Fig. 13A are shown in Supplementary Fig. 2.

### 2.19 Determination of intracellular GSH and GSSG levels

MDA-MB-231 cells (1 × 10^6^/well of a 6-well plate) were incubated for 24 h at 37 °C in 5 % CO_2_ and then treated with vehicle (DMSO, 0.1 %) or test compounds for another 24 h. GSH and GSSG levels were assessed as described [64]. Briefly, cells were scraped in ice-cold 0.1 M potassium phosphate buffer pH 8 supplemented with 8.6 mM EDTA (KPE buffer, 1 mL), washed twice with PBS pH 7.4, and resuspended in 0.2 mL KPE buffer containing sodium fluoride (5 mM), leupeptin (10 μg/mL), soybean trypsin inhibitor (60 μg/mL), phenylmethanesulfonyl fluoride (1 mM), sodium pyrophosphate (2.5 mM), and sodium vanadate (1 mM)). After sonication (3 × 5 s, on ice, 35 % amplitude; Q125, Qsonica), cell lysates were centrifuged (18,000×*g*, 10 min, 4 °C), and supernatants (80 μL) were combined with 50 % aqueous trichloroacetic acid (Carl Roth, #8789.2; 20 μL). Samples were kept on ice for 10 min, and precipitated proteins were removed by centrifugation (9100×*g*, 10 min, 4 °C). For the analysis of GSH, *o*-phthalaldehyde (0.1 mg in methanol) was added to an aliquot of the supernatant (10 μL) in 180 μL KPE buffer to form a fluorescent product. For the analysis of GSSG, GSH in the supernatant (50 μL) was first masked by covalent coupling with *N*-ethylmaleimide (0.4 μmol) for 30 min at room temperature, followed by alkalization with 0.1 N NaOH (180 μL) and reaction of GSSG with *o*-phthalaldehyde (Sigma Aldrich, #79760; 0.1 mg in methanol). After 10 min in the dark at room temperature, the fluorescence (λ_Ex/Em_ = 355/420 nm) was measured using a SpectraMAX iD3 spectrometer (Molecular Devices). Calculation of intracellular GSH and GSSG levels was based on external calibration (0–13.2 μM, 8 concentrations) and normalization to protein content. Protein concentrations were determined in the supernatant after cell lysis using a DC protein assay kit (Bio-Rad Laboratories GmbH).

### 2.20 One-electron oxidation of 3,3′,5,5′-tetramethylbenzidine (TMB)

Test compounds were added to 137 mM sodium acetate buffer pH 5.2 containing TMB (Sigma Aldrich, #860336; 1 mM), 12.5 % (v/v) DMSO, and H_2_O_2_ at the indicated concentrations. The absorbance of the one-electron oxidation product (a diamine-diimine charge transfer complex) was monitored at 652 nm for 10 min using a SpectraMAX iD3 spectrometer (Molecular Devices).

### 2.21 Electron paramagnetic resonance (EPR) spin trapping

Vehicle (methanol, 10 %) or test compounds (0.5 mM) were added to an aqueous solution of 5,5-dimethyl-1-pyrroline *N*-oxide (DMPO; Cayman, #10006436; 100 mM) and H_2_O_2_ (5 mM) at pH 2.8, which was supplemented with aqueous glycerol pH 2.8 (final concentration: 5 %) to reach in total 200 μL. The reaction mixture was transferred to a capillary tube and subjected to EPR measurements. EPR spectra were recorded in Quartz 3 mm OD J-Young style fused silica tubes (ATS Life Sciences Wilmad, Vineland, NJ) at −15 °C using a Magnettech MS-5000 X-band benchtop EPR spectrometer (Bruker, Billericia, MA) equipped with a variable temperature unit [65]. EPR spectrometer settings (modified according to Ref. [66]): modulation frequency, 100 kHz, X-band microwave frequency, 9.5 GHz; microwave power, 10 mW; modulation amplitude, 0.1 mT; magnetic field sweep, 330–345 mT; scan time 120 s.

### 2.22 Cell-free oxidation of NADPH

Vehicle (DMSO, 1 %) or test compounds (0.1 mM) were added to an aqueous solution of NADPH (0.5 mM) and, where indicated, H_2_O_2_ (37 mM). Changes in NADPH levels were monitored for 20 min by measuring the absorbance at 340 nm using a SpectraMAX iD3 spectrometer (Molecular Devices).

### 2.23 Cyclic voltammetry

Electrochemical measurements were performed as previously described [29]. In brief, the SP-150 potentiostat (BioLogic, Seyssinet-Pariset, France) was equipped with a conventional three-electrode cell, a glassy carbon working electrode, an Ag/AgCl reference electrode in saturated NaCl solution, and a 0.5 × 37 mm/1 mm × 37 mm platinum wire counter electrode (Alibaba Group, Shenzhen, China). The test compounds (1 mM) were dissolved in a solution of the supporting electrolyte tetrabutylammonium hexafluorophosphate (0.5 M; TCI, Tokyo, Japan) in anhydrous solvents [dichloromethane (TCI) or DMSO (Thermo Fisher Scientific)] in preheated volumetric flasks and transferred to the microcell by syringe. To create oxygen-free conditions, the solution was purged with argon, and electrochemical studies were performed under an argon atmosphere. Five scans (cycles) were performed at a scan rate of 100 mV/s for each measurement. The anodic and cathodic standard potentials were calculated with EC-Lab® V11.31 (BioLogic) using ferrocene (2 mM) as an internal standard.

### 2.24 Cellular NADPH/NADP^+^ assay

MDA-MB-231 cells (5 × 10^5^/well of a 6-well plate) were incubated for 24 h at 37 °C in 5 % CO_2_ and then treated with vehicle (DMSO, 0.1 %) or test compounds (1 μM) for another 24 h. Cells were harvested and an aliquot (8 × 10^4^ cells) was resuspended in 50 μL PBS pH 7.4 and lysed by addition of 50 μL of 0.2 M NaOH containing 1 % (v/v) dodecyltrimethylammonium bromide (DTAB) (Cayman, #37009). The total amount of NADPH and NADP^+^ was determined using the NADP/NADPH-Glo™ Assay Kit (#G9071, Promega, Madison, WI) following the manufacturer's instructions.

### 2.25 Mitochondrial membrane potential

MDA-MB-231 cells (5 × 10^5^/25 cm^2^) were cultured for 24 h at 37 °C in 5 % CO_2_ before treatment with vehicle (DMSO, 1 %), **1** (0.3 μM), or **1** (0.3 μM) plus Fer-1 (3 μM). Cells were washed three times with PBS pH 7.4 and detached by trypsinization. Aliquots (1–3 × 10^6^) were washed again with PBS pH 7.4, and cell pellets (300×*g*, 5 min) were incubated for 25 min in the dark at 37 °C in complete cell culture medium (500 μL) supplemented with JC-1 (0.5 μg/mL). Controls include i) untreated cells, which were not exposed to test compounds or JC-1, but were treated with cell culture medium (500 μL) at 37 °C for 25 min, ii) uncoupled cells, which were treated with FCCP (10 μM) and JC-1 (0.5 μg/mL) in cell culture medium (500 μL) at 37 °C for 25 min, and iii) ATP synthase-blocked cells, which were first incubated in cell culture medium (500 μL) supplemented with JC-1 (0.5 μg/mL) at 37 °C for 25 min and then treated with oligomycin (0.3 μM) at 37 °C for 5 min. The pelleted cells (300×*g*, 5 min) were washed three times with PBS pH 7.4 (1 mL) and resuspended in PBS pH 7.4 (0.3 mL). The mitochondrial membrane potential was analyzed using a LSRFortessa™ Cell Analyzer (BD Biosciences, Becton, NY) by determining the fluorescence intensity ratio of JC-1 aggregates (Ex/Em = 488/582 nm) and JC-1 monomers (Ex/Em = 488/530 nm), calculated for each cell and averaged. Data were processed using FlowJo (BD Biosciences). The gating strategy is shown in Supplementary Fig. 1B.

### 2.26 Cellular catalase activity

MDA-MB-231 cells (5 × 10^5^/well) were seeded in 6-well plates and treated after 24 h at 37 °C in 5 % CO_2_ with vehicle (DMSO, 0.1 %), RSL3 (1 μM), **1**, **2**, or **3** (0.3 μM) for another 24 h. Cells were harvested and the cell pellet was homogenized and sonicated in 1 mL of 1 × Assay buffer per 2 × 10^6^ cells. Total catalase activity was determined using a catalase colorimetric activity kit (Thermo Fisher Scientific, #EIACATC) following the manufacturer's instructions.

### 2.27 Statistical analysis

Data were analyzed using Microsoft 365 (Microsoft, Redmond, WA) and are reported as the mean ± SEM of *n* independent experiments. Statistical calculations on non-transformed or logarithmized data were performed with GraphPad Prism 9.5 (GraphPad Software, San Diego, CA) by one-way or two-way ANOVA for independent or correlated samples, followed by Dunnett's or Sidak's *post hoc* tests, or by two-tailed Student *t*-tests for unpaired samples. No data were excluded, unless experimental controls were not successful, resulting in the exclusion of the independent dataset. The *α* level was set at 0.05, and *P* values < 0.05 were considered statistically significant. Volcano plots show the mean difference of changes in absolute intensities and the negative log_10_ (adjusted *P* value). Adjusted *P* values were calculated by two-tailed, multiple unpaired Student *t*-tests with correction for multiple comparisons using a two-stage linear step-up procedure by Benjamini, Krieger, and Yekutieli (false discovery rate 5 %). The LC_50_ values were determined through non-linear regression analysis of normalized responses using a 3-parameter logistic regression equation with GraphPad Prism 10.0.0 software (GraphPad Software). Vector plots were generated using GraphPad Prism 10.0.0 (GraphPad Software), except for radar plots, extracted chromatograms, and cyclovoltagrams, which were prepared using OriginPro 2020 (OriginLab, Northampton, MA). Heat maps were generated using Microsoft Excel (Microsoft, Redmond, WA), and chemical structures were drawn using KingDraw 20.0 (KingAgroot, Qingdao, China).

## 3 Results

### 3.1 SCs induce cell death in therapy-resistant cancer cells

**Fig. 1:**
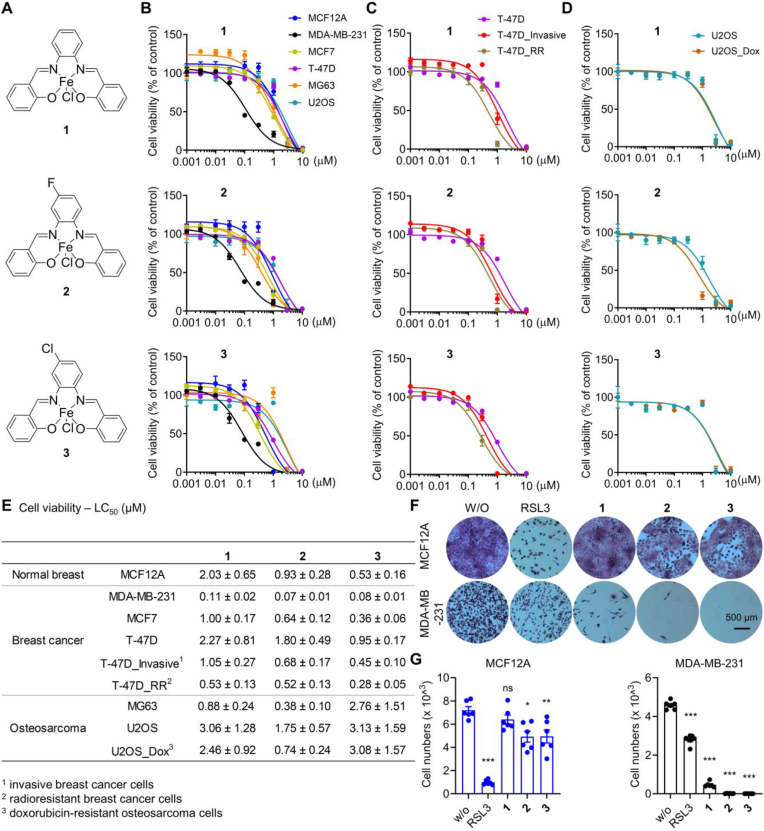
Induction of cell death in therapy-resistant breast cancer cells by SCs. A. Chemical structures of SCs **1**–**3**. B-E. Cells seeded in 96-well plates were incubated after 24 h with vehicle (‘w/o’, 0.5 % DMSO), **1**, **2**, or **3** (0.001–10 μM) for 48 h and the cellular dehydrogenase activity was analyzed as measure of cell viability (MTT assay). B. Non-malignant human breast cells (MCF12A: 1 × 10^4^) and breast cancer cells (MDA-MB-231: 2 × 10^4^; MCF7: 1 × 10^4^, T-47D: 1.5 × 10^4^). C. Invasive T-47D cells (T-47D_invasive: 2 × 10^4^) and radioresistant T-47D cells (T-47D_RR: 7.5 × 10^3^). D. Human U2OS (1.5 × 10^4^) and MG63 (7.5 × 10^3^) osteosarcoma cells and doxorubicin-resistant U2OS cells (U2OS_Dox: 1.5 × 10^4^). E. LC_50_ values calculated by non-linear regression analysis from the data shown in B-D. F, G. Crystal violet-stained colonies of MCF12A or MDA-MB-231 cells (1 × 10^4^/well in a 6-well plate) treated every 2 days for 10 days with vehicle (‘w/o’, 0.1 % DMSO), RSL3 (0.03 μM), **1**, **2**, or **3** (0.03 μM each) in fresh culture medium. F) Phase-contrast microscopic images. G. Cell number determined by automated cell tracking from two technical replicates per independent sample. Data are expressed as mean ± SEM from n = 3 independent experiments. *P < 0.05, **P < 0.01, ***P < 0.001 compared to vehicle control (G); repeated measures one-way ANOVA + Dunnett's *post hoc* tests. (For interpretation of the references to color in this figure legend, the reader is referred to the Web version of this article.)

SCs are potent inducers of cell death in several cancer cell lines [29,31,33,42,43,67,68], including cisplatin-resistant cells [31,42,68]. To further explore the therapeutic potential of SCs in the treatment of therapy-resistant cancers, we investigated the cytotoxic activity of [Chlorido (Fe(III)-salophene)] (**1**) and its two close structural derivatives [Chlorido (Fe(III)-salophene)]-F (**2**) and [Chlorido (Fe(III)-salophene)]-Cl (**3**) (Fig. 1A) on malignant and therapy-resistant human breast cancer and osteosarcoma cell lines in comparison to normal cells (Fig. 1B–E). Given the strong dependence of ferroptosis on cell density, we first monitored the proliferation rates of individual cell lines (Supplementary Figs. 3A–C) and then adjusted the number of cells seeded to achieve comparable cell densities over the 24 h treatment period. SCs potently suppressed the mitochondrial activity (measured by MTT assay) of metastatic triple-negative MDA-MB-231 breast cancer cells with a mesenchymal-like phenotype (lethal concentration 50 (LC_50_) = 0.07–0.11 μM; Fig. 1B, **E**). Other cell lines tested were less susceptible (LC_50_ = 0.28–3.13 μM), including epithelial breast cancer cell lines (MCF7 and T-47D), epithelial-like non-malignant breast cells (MCF12A), fibroblast-like MG63 osteosarcoma cells, mesenchymal-like U2OS osteosarcoma cells (Fig. 1B–E), epithelial-like HepG2 hepatocellular carcinoma cells, and epithelial-like A549 lung carcinoma cells (Supplementary Fig. 3C), with potency increasing from **1** < **2** < **3** (Fig. 1E). T-47D cells selected for invasiveness or radioresistance were killed equally or even more efficiently (Fig. 1C, **E**), and comparable results were obtained in doxorubicin-resistant U2OS cells (Fig. 1D, **E**). The lethal activity of SCs exceeds the cytotoxic potency of routine ferroptosis inducers, such as the GPX4 inhibitor RSL3 (LC_50_ = 0.18 μM) and the system Xc^−^ inhibitor erastin (LC_50_ = 2.27 μM) (Supplementary Fig. 3D), and does not correlate with the cytotoxic activity of RSL3 across cell lines (Supplementary Figs. 3C–E). The higher selectivity of SCs towards MDA-MB-231 breast cancer cells over non-cancerous cells was confirmed by crystal violet staining (Fig. 1F) and counting of stained cells after removal of dead, detached cells by washing (Fig. 1G). Together, SCs preferentially induce cell death in metastatic triple-negative breast cancer cells over other cancer and non-cancer cell lines and are equally or even more active against chemoresistant, radioresistant or invasive cancer cells as compared to the parental cells.

**Fig. 2:**
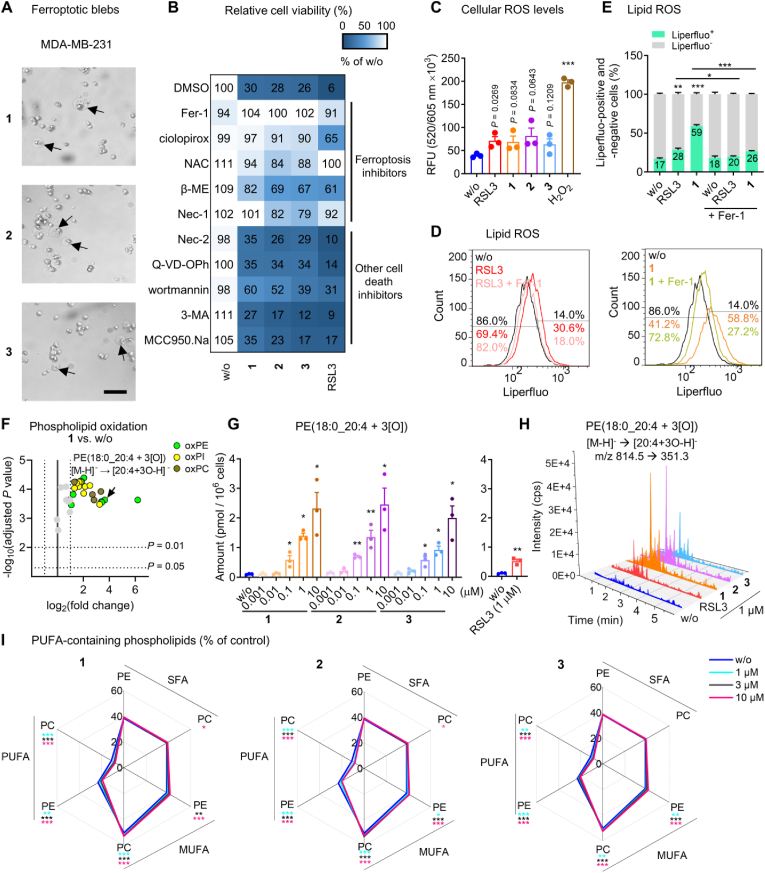
SCs trigger excessive phospholipid peroxidation in triple negative breast cancer cells. A, B. MDA-MB-231 cells (2 × 10^4^/well of a 96-well plate) were treated with vehicle (‘w/o’, 0.5 % DMSO), **1**, **2**, **3** (A: 1 μM each; B: 0.1 μM each) or RSL3 (1 μM) in the presence or the absence of cell death inhibitors for 48 h before cell viability was determined. A. Phase contrast microscopic images (scale bar: 500 μm). B. Inhibition of cell death induction by ferroptosis inhibitors but not by other cell death inhibitors. Ferroptosis inhibitors: Fer-1 (3 μM), ciclopirox (0.25 μM), *N*-acetyl-*l*-cysteine (NAC, 2.5 mM), β-mercaptoethanol (β-ME, 200 μM), necrostatin-1 (Nec-1, 40 μM; also inhibits necroptosis); necroptosis inhibitor: necrostatin-2 (Nec-2, 10 μM), apoptosis inhibitor: Q-VD-OPh (20 μM); autophagy inhibitors: wortmannin (1 μM), 3-methyladenine (3-MA, 1 mM); pyroptosis inhibitor: MCC950.Na (1 μM). C. Cellular ROS formation of MDA-MB-231 cells (2 × 10^4^/well of a 96-well plate) treated with vehicle (‘w/o’, 0.5 %), RSL3 (1 μM), **1**, **2**, **3** (1 μM each), or H_2_O_2_ (1 mM) for 2 h. D, E. Flow cytometric analysis of liperfluo-stained lipid peroxides in MDA-MB-231 cells (5 × 10^5^/well of a 6-well plate) treated with vehicle (‘w/o’, 0.5 % DMSO), RSL3 (1 μM), **1** (1 μM), or **1** together with Fer-1 (3 μM) for 6 h. D. Histogram showing cell number as a function of liperfluo staining. E. Quantitative analysis of liperfluo-positive and -negative cells based on the data from panel D. F–I. MDA-MB-231 cells (3.12 × 10^6^/75 cm^2^) were treated with vehicle (‘w/o’, 0.1 % DMSO), **1**, **2**, **3** (1 μM each, unless otherwise stated), or RSL3 (1 μM) for 2 h, and oxidized and non-oxidized PE, PC, and PI species were analyzed by UPLC-MS/MS. F. Volcano plot showing the log2 of fold-change in the amount of (per)oxidized PC, PE, and PI species relative to vehicle control and the negative log10 (adjusted *P* value) calculated vs. vehicle control; two-tailed multiple unpaired Student t tests with correction for multiple comparisons (false discovery rate 5 %). G. Amount of PE(18:0_20:4 + 3[O]). H. Extracted chromatograms based on the fragmentation of [PE(18:0_20:4 + 3[O]-H]^-^ to [20:4 + 3[O]-H]^-^. I. Percentage changes in the proportion of SFA-, MUFA-, and PUFA-containing PC and PE. Data are expressed as mean ± SEM from n = 3 independent experiments. *P < 0.05, **P < 0.01, ***P < 0.001 compared to vehicle control or indicated by bars; repeated measures one-way + Dunnett's tests on log-transformed data (G) or repeated measures two-way ANOVA + Dunnett's (I) or Tukey's post hoc test (E) or two-tailed unpaired Student t-test (C, G, RSL3).

### 3.2 Cell death induction by SCs is preceded by excessive phospholipid peroxidation

Because of the high sensitivity of triple-negative breast cancer cells to SCs (Fig. 1B, ***E*-G**), we selected MDA-MB-231 cells for further mechanistic studies on cell death induction. When treated with SCs, MDA-MB-231 cells acquire an advanced ferroptotic phenotype with characteristic large blebs (Fig. 2A; blebs indicated by arrows). Compensation experiments with selective cell death inhibitors indicate that ferroptosis is the predominant cell death program induced (Fig. 2B, Supplementary Fig. 4A). Thus, the cytotoxic activity of SCs was efficiently suppressed by ferroptosis inhibitors that scavenge lipid radicals (Fer-1), chelate iron (ciclopirox), or increase the intracellular redox tonus (*N*-acetyl-*l*-cysteine, NAC; β-mercaptoethanol, β-ME). Selective inhibition of apoptosis (Q-VD-OPh), necroptosis (necrostatin-2, Nec-2), pyroptosis (MCC950.Na), and autophagy (wortmannin; 3-methyladenine, 3-MA) was instead not protective (Fig. 2B) and only necroptosis but not apoptosis markers were elevated by trend (Supplementary Fig. 4B). Similar profiles were observed for the ferroptosis inducer RSL3 (Fig. 2B, Supplementary Fig. 4A). Note that SCs have recently been reported to induce necroptosis in leukemia cells based on studies with necrostatin-1 (Nec-1) [28], which also protects MDA-MB-231 cells from SC-induced death (Fig. 2B, Supplementary Fig. 4A). However, Nec-1 is not selective for necroptosis, but also suppresses ferroptosis [69], and we consider the latter to be dominant in MDA-MB-231 cells, given the failure of the more selective necroptosis inhibitor Nec-2 to prevent cell death (Fig. 2B, Supplementary Fig. 4A).

The induction of ferroptosis in breast cancer cells by **1** is associated with an increase in total ROS levels, comparable to the treatment with RSL3 (Fig. 2C). In addition, **1–3** evoked marked lipid peroxidation that exceeds the response to the GPX4 inhibitor RSL3 and is attenuated by Fer-1 [70], as shown by flow cytometric analysis of lipid hydroperoxides stained with liperfluo (Fig. 2D, **E**). Detailed profiling of phospholipids by targeted redox lipidomics revealed a strong (per)oxidation of arachidonic acid (20:4) and adrenic acid (22:4), especially in phosphatidylethanolamines (PEs) and phosphatidylinositols (PIs) and less in phosphatidylcholines (PCs) (Fig. 2F-**H**, Supplementary Fig. 5, Supplementary Fig. 7A), with **1** and **2** again being superior to RSL3 (Fig. 2G, **H**, Supplementary Fig. 6, Supplementary Fig. 7A). Among the most abundant and strongly up-regulated oxidized phospholipids were 2[O] and 3[O] species derived from PE(18:0_20:4) and PI(18:0_20:4) (Supplementary Fig. 5, Supplementary Fig. 6). The kinetic settings were pre-optimized for RSL3-treated MDA-MB-231 cells by monitoring the formation of 1[O], 2[O], and 3[O] PE and PC species over a period of 24 h. Prominent phospholipid (hydro)peroxidation was evident at 2 h and then decreased to baseline within 4–24 h (Supplementary Fig. 7B, Supplementary Fig. 8). As expected, Fer-1 efficiently blocked RSL3-induced PE and PC (per)oxidation (Supplementary Figs. 7C–D). Since only a small fraction of membrane PUFAs is peroxidized during ferroptosis [14], the fatty acid composition of major membrane phospholipids (i.e., PC and PE) (Supplementary Figs. 7E–F) barely changed upon short-term treatment with RSL3 (Supplementary Figs. 7G–H, Supplementary Fig. 9-**10**). Instead, the more active SC complexes depleted cells of PUFAs within 2 h, resulting in a relative enrichment of monounsaturated fatty acid (MUFA)-containing phospholipids (Fig. 2I, Supplementary Fig. 9-**10**). Together, SCs induce efficient (per)oxidation of major membrane phospholipids, outperforming established ferroptosis inducers.

### 3.3 Lipophilic SCs catalyze phospholipid peroxidation

**Fig. 3:**
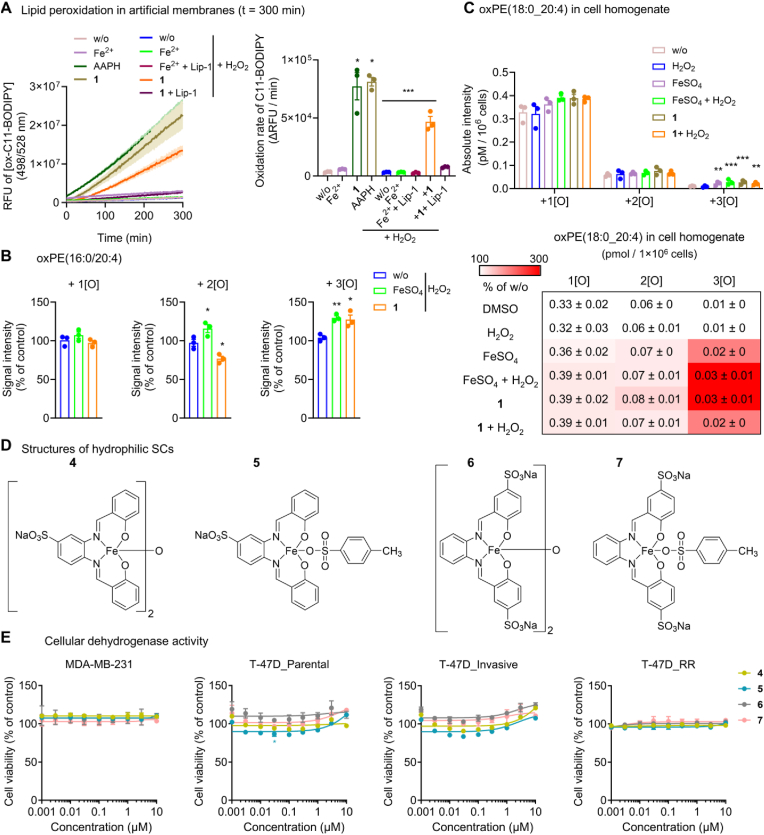
Lipophilic SCs enable efficient phospholipid peroxidation under cell-free conditions. A. Time-dependent analysis of PC peroxidation in artificial membranes upon incubation with AAPH (10 μM), FeSO_4_ (10 μM), or **1** (10 μM) in the presence or the absence of H_2_O_2_ (10 μM) and liproxstatin-1 (Lip-1, 1 μM). The bar chart shows the difference in peroxidation at 300 min. B, C Quantitative analysis of phospholipid oxidation products by UPLC-MS/MS. B. Oxidation products of PE(16:0_20:4) in mixed micelles in the presence of H_2_O_2_ (100 μM) after incubation with vehicle (‘w/o’, 1 % DMSO), FeSO_4_ (100 μM), or **1** (100 μM) for 2 h at 37 °C. C. Oxidation products of PE(18:0/20:4) in MDA-MB-231 homogenates (from 3 × 10^6^ cells) treated with vehicle (‘w/o’, 1 % DMSO), FeSO_4_ (100 μM), FeSO_4_ + H_2_O_2_ (100 μM), or **1** (100 μM) for 2 h at 37 °C. D. Chemical structures of the hydrophilic SCs **4**–**7**. E. Cell viability of MDA-MB-231 cells (2 × 10^4^), T-47D cells (‘T-47D_parental’, 1.5 × 10^4^), invasive T-47D cells (‘T-47D_invasive’, 2 × 10^4^), and radioresistant T-47D cells (‘T-47D_RR’, 7.5 × 10^3^) seeded in 96-well plates and incubated (after 24 h) with vehicle (DMSO, 0.5 %) or **4**–**7** for 48 h. Data are expressed as mean ± SEM from n = 3 independent experiments. **P < 0.01, ***P < 0.001 compared to vehicle control or as indicated by bars; ordinary one-way ANOVA + Dunnett's *post hoc* tests (A, B) or ordinary two-way ANOVA + Dunnett's *post hoc* tests on log-transformed data (C) or repeated two-way ANOVA + Dunnett's *post hoc* tests (E).

Given the lipophilic nature of SCs, which is expected to position the redox-active iron core close to membranes, we speculated that SCs would propagate lipid peroxidation, as described for mobile iron [71]. Indeed, **1** efficiently induced phospholipid peroxidation in liposomes composed of egg PC, alone and in combination with H_2_O_2_, being more effective than Fe^2+^/H_2_O_2_ and reaching the peroxidation efficacy of the free radical-generating azo compound 2,2′-azobis (2-amidinopropane) dihydrochloride (AAPH) (Fig. 3A). Liposome peroxidation is inhibited by the lipophilic radical trap liproxstatin-1. Studies on isolated PE(16:0_20:4) (Fig. 3B) and MDA-MB-231 homogenates (Fig. 3C), either in presence or absence of H_2_O_2_, confirm that **1** catalyzes the incorporation of three (rather than two) oxygens into PUFA-containing PE. To investigate whether SCs need to be lipophilic to induce ferroptosis, we synthesized hydrophilic analogs (**4**, **5**, **6**, and **7**) with one or two sulfonate substituents at the otherwise identical Fe(III)-salophen core (Fig. 3D). None of these compounds suppressed the viability of parental, invasive, and resistant human breast cancer cell lines (Fig. 3E), indicating that the salophen-complexed iron, combined with the overall lipophilicity, renders SCs to potent ferroptosis inducers. Note that we cannot rule out differences in the cellular uptake between **1**–**3** and **4**–**7**.

**Fig. 4:**
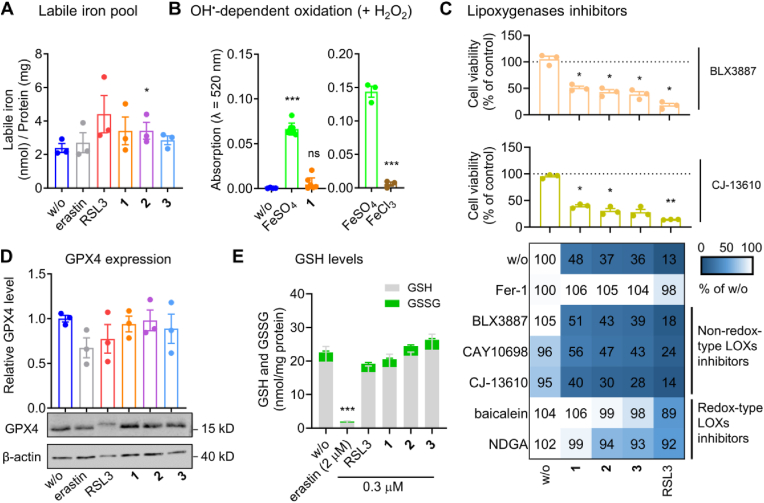
The cytotoxic mechanism of SCs is independent from major cellular pathways regulating ferroptosis. A. Labile iron content of MDA-MB-231 cells (1 × 10^6^/25 cm^2^) treated with vehicle (‘w/o’, 0.1 % DMSO), erastin (3 μM), RSL3 (3 μM), **1**, **2**, or **3** (0.3 μM each) for 24 h. B. Extent of OH-radical-dependent oxidation of salicylic acid (0.52 mM) by vehicle (‘w/o’, H_2_O), FeSO_4_ (left panel: 0.1 mM; right panel: 0.25 mM), FeCl_3_ (0.25 mM), or **1** (0.1 mM) in the presence of H_2_O_2_ (left panel: 37 mM; right panel: 225 mM) within 30 min at room temperature. C. Non-redox-type 5-, 12-, and 15-LOX inhibitors do not interfere with cell death induction by SCs. MDA-MB-231 cells (2 × 10^4^/well of a 96-well plate) were incubated with vehicle (‘w/o’, 0.5 % DMSO), RSL3 (1 μM), **1**, **2**, or **3** (0.1 μM) for 48 h in the presence or the absence of Fer-1 (3 μM) or LOX inhibitors before cell viability was determined. Non-redox-type LOX inhibitors: 15-LOX-1 inhibitor BLX3887 (3 μM), 12-LOX inhibitor CAY10698 (10 μM), 5-LOX inhibitor CJ-13610 (1 μM). Redox-type LOX inhibitors: pan-LOX inhibitors baicalein (3 μM) and NDGA (3 μM). D. GPX4 protein levels of MDA-MB-231 cells (5 × 10^5^/well of a 6-well plate) incubated with vehicle (‘w/o’, 0.1 % DMSO), erastin (2 μM), RSL3 (1 μM), **1**, **2**, or **3** (0.1 μM each) for 24 h. E. GSH and GSSG levels of MDA-MB-231 cells (1 × 10^6^/well of a 6-well plate) incubated with vehicle (‘w/o’, 0.1 % DMSO), erastin (2 μM), RSL3 (0.3 μM), **1**, **2**, or **3** (0.3 μM each) for 24 h. Data are expressed as mean ± SEM from n = 3 (except n = 8 for B, left panel) independent experiments. *P < 0.05, ***P < 0.001 compared to vehicle (A, B, D, E) or the inhibitor control (C); repeated measures one-way ANOVA + Dunnett's *post hoc* tests (D) on log-transformed data (A, C) or ordinary one-way ANOVA + Dunnett's *post hoc* tests (B, left panel) or two-tailed unpaired Student t-test (B, right panel).

### 3.4 Other important features of ferroptosis are not manipulated by SCs

Motivated by the promising results of SCs on lipid peroxidation, we systematically investigated the underlying molecular mechanisms, focusing on established key players in ferroptosis. Since the complexes used in this study are Fe(III)-containing SCs, we speculated that the compounds might either be Fenton-active or increase the mobile Fe(II) pool, thereby generating hydroxide (OH) radicals from hydrogen peroxide (H_2_O_2_) to attack PUFAs. However, **1** did not readily increase labile iron levels (which rather excludes an effect on iron metabolism or the release of iron from the complexes) (Fig. 4A), nor did it generate OH radicals, unlike FeSO_4_ (Fig. 4B, Supplementary Figs. 11A–B), as shown by a colorimetric indicator reaction (Supplementary Fig. 11C). SC-induced lipid peroxidation does also not involve lipoxygenases (LOXs), as neither selective, non-redox-active 15-LOX-1 (BLX3887), 12-LOX (CAY10698), nor 5-LOX inhibitors (CJ-13610) attenuated the decrease in cellular dehydrogenase activity (Fig. 4C). Note that redox-active LOX inhibitors (baicalein, NDGA) prevented SC-induced cell death, which we attribute to their radical scavenging activity rather than interference with LOX activity [72,73]. Furthermore, SCs did not substantially affect GPX4 expression (Fig. 4D) or glutathione (GSH) and glutathione disulfide (GSSG) levels (Fig. 4E), raising the question of the peroxidation-promoting mechanism.

### 3.5 SCs initiate a redox cycle in the presence of H_2_O_2_ that consumes NADPH

**Fig. 5:**
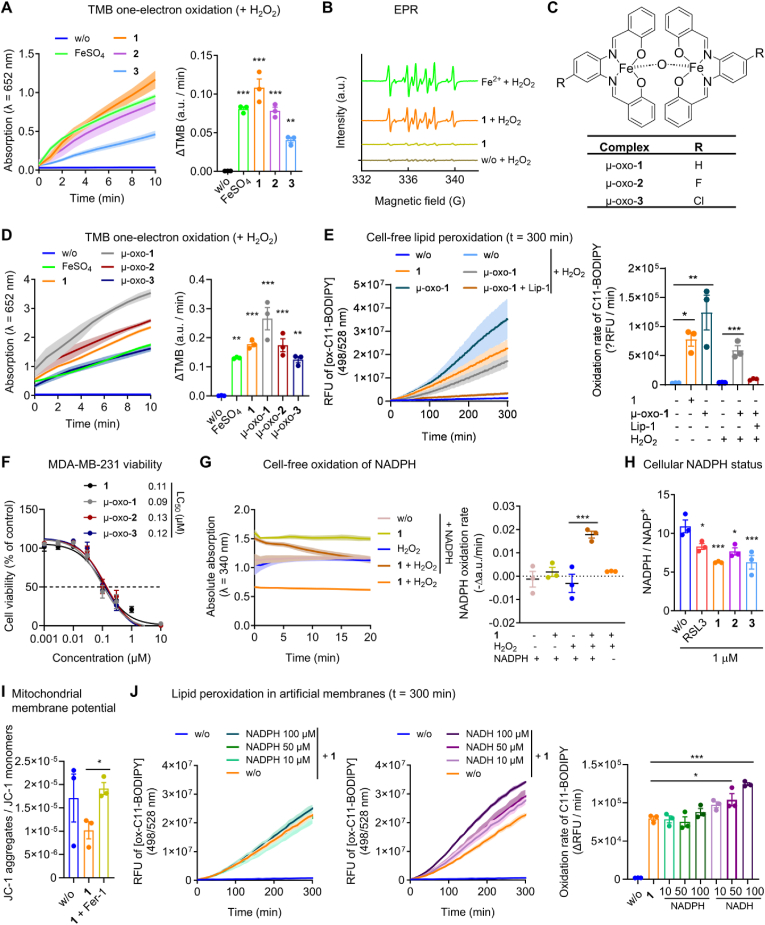
SCs catalyze H_2_O_2_-dependent redox reactions that deplete cells of NADPH. A. One-electron oxidation of TMB (1 mM) by vehicle (‘w/o’, 1 % DMSO), FeSO_4_ (1 mM), **1**, **2**, or **3** (0.1 mM each) in the presence or the absence of H_2_O_2_ (25 mM). The bar chart shows the oxidation rate (change in absorbance per min). B. EPR spectra showing unpaired electron signals (characteristic of DMPO-OCH_3_ adduct radicals) generated by the decomposition of H_2_O_2_ (5 mM) by FeSO_4_ (0.5 mM) or **1** (0.5 mM) with methanol (10 %) as vehicle. Brown line: DMPO + H_2_O_2_; golden line: **1** + DMPO (w/o H_2_O_2_); orange line: 1 + DMPO + H_2_O_2_; green line: Fe^2+^ + DMPO + H_2_O_2_. C. Chemical structures of the μ-oxo-SCs μ-oxo-**1**, μ-oxo-**2**, and μ-oxo-**3**. D. One-electron oxidation of TMB (1 mM) by vehicle (‘w/o’, 1 % DMSO), FeSO_4_ (0.15 mM), **1** (0.15 mM), μ-oxo-**1**, μ-oxo-2, or μ-oxo-3 (0.075 mM each) in the presence or the absence of H_2_O_2_ (25 mM). The bar chart shows the oxidation rate (change in absorption per min). E. Time-dependent analysis of PC peroxidation in artificial membranes upon incubation with **1** (10 μM, identical data as shown in Fig. 3A) or μ-oxo-**1** (10 μM) in the presence or the absence of H_2_O_2_ (10 μM) and liproxstatin-1 (Lip-1, 1 μM). The bar chart shows the difference in peroxidation at 300 min. F. Cell viability of MDA-MB-231 cells (2 × 10^4^/well of a 96-well plate) incubated with vehicle (DMSO, 0.5 %), **1** (identical data as shown in Fig.1B), μ-oxo-**1**, μ-oxo-**2**, or μ-oxo-**3** for 48 h. G. Photometric analysis of the oxidation of NADPH (0.5 mM) by H_2_O_2_ (37 mM) and vehicle (‘w/o’, 1 % DMSO) or **1** (0.1 mM) or in the absence of H_2_O_2_. The median and single value plot indicates the oxidation rate (change in absorption per min). H. Ratio of NADPH to NADP^+^ in MDA-MB-231 cells (5 × 10^5^/well of a 6-well plate) treated with vehicle (‘w/o’, 0.1 % DMSO), RSL3 (1 μM), **1**, **2**, or **3** (1 μM each) for 24 h. I. Ratio of JC-1 aggregates to JC-1 monomers as measure for the mitochondrial membrane potential in MDA-MB-231 cells treated with vehicle (‘w/o’, 1 % DMSO), **1** (0.3 μM), or **1** (0.3 μM) plus Fer-1 (3 μM) for 24 h. J. Time-dependent analysis of PC peroxidation in artificial membranes upon incubation with **1** (10 μM) in the presence or the absence of NADPH and NADH. Data are expressed as mean ± SEM from n = 3 independent experiments. *P < 0.05, **P < 0.01, ***P < 0.001 compared to vehicle control or as indicated by bars; two-sided unpaired Student t-test (I) or ordinary one-way ANOVA + Dunnett's *post hoc* tests. (For interpretation of the references to color in this figure legend, the reader is referred to the Web version of this article.)

SCs may catalyze redox reactions that reduce the antioxidant capacity of cells without generating free OH radicals. To explore this hypothesis, we monitored the one-electron oxidation of 3,3′,5,5′-tetramethylbenzidine (TMB) by H_2_O_2_ to a radical cation that forms a charge-transfer complex that absorbs at 652 nm (Supplementary Fig. 12) [74]. SCs strongly increase TMB oxidation (**1** > **2** > **3**), comparable to free Fe^2+^ (Fig. 5A), indicating that they are capable of accelerating one-electron transfer reactions. We then determined the identity of the radicals formed during the SC-catalyzed decomposition of H_2_O_2_ by electron paramagnetic resonance (EPR) spectroscopy using DMPO as a spin trap reagent. Compound **1** and free Fe^2+^ generate radicals, preferentially (secondary) methoxy radicals [75], in the presence of H_2_O_2_ and methanol as vehicle (Fig. 5B). Likely in compensation, the expression and activity of antioxidant enzymes, i.e., catalase, superoxide dismutase (SOD)1, thioredoxin reductase (TXNRD)1, and glutathione peroxidase (GPX)1, tended to be upregulated, reaching significance for TXNRD1 (Supplementary Fig. 13A, **B**).

Electrochemical studies were performed to gain more detailed insights into the one-electron transfer mechanism of SCs. As previously shown for **1** by cyclic voltammetry [29,76,77] and confirmed here for **2** and **3**, monomeric SCs form a reversible iron (III/II) redox pair under inert conditions (i.e., dichloromethane (DCM) under argon (Ar)) (Supplementary Fig. 14A, **B**, Table S6). In the presence of oxygen, Fe(III) is still reduced to Fe(II), but is no longer reoxidized to Fe(III) in monomeric SCs. Instead, an additional redox pair appears (Supplementary Fig. 14A, **B**, Table S6), which has previously been ascribed to dimeric μ-oxo-[(Fe(III)-4-halogen-salophen)]_2_ (μ-oxo-**1**, μ-oxo-**2**, and μ-oxo-**3**) (Fig. 5C) [29] and indeed reflects the reduction and oxidation peaks of the corresponding μ-oxo standards (Supplementary Fig. 14A, **B**, Table S6). Comparable results were obtained in DMSO as solvent, which was used to distinguish the peaks indicating the redox pairs of μ-oxo species from those representing the reduction of oxygen to superoxide (Supplementary Fig. 14C, **D**, Table S7). Note that **1** exchanges the axial ligand in DMSO and that μ-oxo species are partially decomposed to form monomeric SCs [29]. Taken together, SCs have a high affinity for oxygen after reduction of Fe(III) to Fe(II) and are converted to oxo-bridged dimeric Fe(III) complexes, which can undergo an independent redox cycle, but might also disintegrate back to monomeric complexes. In support of this hypothesis, μ-oxo-**1** is comparably effective to **1** in oxidizing TMB (Fig. 5D), inducing phospholipid peroxidation in liposomes in vitro (Fig. 5E), and triggering cell death in breast cancer cells (Fig. 5F).

To explore whether this redox cycle might deplete cells of essential cofactors, we exemplarily determined the effect of **1** on the redox balance between NADPH and NADP^+^. Compound **1** strongly enhanced NADPH oxidation by H_2_O_2_ in a cell-free assay (Fig. 5G), being more efficient than non-chelated Fe(II) (Supplementary Fig. 14E). In addition, SCs markedly decreased the NADPH/NADP^+^ ratio in breast cancer cells (Fig. 5H) and lowered the mitochondrial membrane potential (which is fueled by redox equivalents such as NADH), an effect that is prevented by the lipophilic radical trap Fer-1 (Fig. 5I). Although we do not consider the catalytic activity of SCs to be limited to NAD(P)H, the complexes seem to be selective to some extent, as suggested by their apparent failure to efficiently oxidize GSH and decrease the cellular ratio of GSH to GSSG (Fig. 4E). Finally, we investigated whether NADPH or NADH, in addition to being degraded by **1**, also contributes to the reductive activation of SCs. Indeed, NADH (and by trend NADPH) increased the rate of artificial membrane peroxidation by **1** (Fig. 5J), suggesting that a reduction of SC-bound Fe(III) to Fe(II) sustains the redox cycle.

## 4 Discussion

**Fig. 6:**
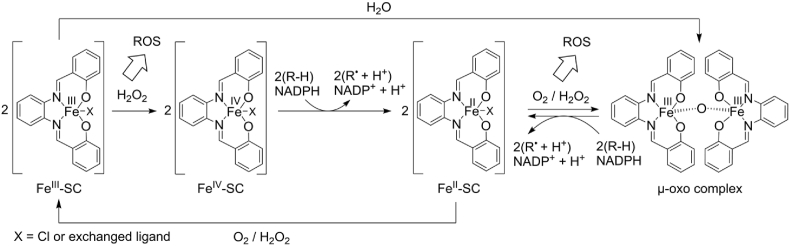
Proposed catalytic mechanism of SCs for the (per)oxidation of phospholipid-bound PUFAs and redox cofactors. Initiation: H_2_O_2_ oxidizes Fe^III^ in SCs to a hyperoxidative Fe^IV^ state, allowing one- or two-electron oxidations of redox substrates, such as TMB or endogenous factors, including the allylic positions of PUFAs in membrane phospholipids (*R*–H) or NADPH, thereby yielding Fe^II^-SC. NADPH is a central cofactor in the control of redox homeostasis that keeps ferroptosis at bay, but also has pleiotropic other functions, some of which are related to cell death programs other than ferroptosis. Alternatively, FeIII–SCs may spontaneously dimerize to a minor extent in aqueous solutions to form μ-oxo complexes [29]. Propagation: Fe^II^-SC then undergoes a redox cycle initiated by the oxidation to μ-oxo complexes with a binuclear Fe^III^ center, which in turn allows one- or two-electron oxidation of redox substrates in the course of regenerating monomeric Fe^II^-SC. Reactive oxygen species (not necessarily OH-radicals) are formed from O_2_ or H_2_O_2_ during these multiple oxidation steps, which also involve the formation of secondary radicals.

We show here that SCs are highly effective against aggressive and metastatic human triple-negative MDA-MB-231 breast cancer cells, which as mesenchymal cells are susceptible to ferroptosis per se, apparently due to adjustments in PUFA metabolism [78]. While non-malignant and epithelial breast cancer cells are less affected, cell lines with acquired therapy resistance are not specifically protected from SCs. These findings in breast and osteosarcoma cancer cells add to a large number of studies demonstrating the efficacy of SCs against therapy-resistant cancer cells, both in vitro and in vivo [31,33,36,38,39,42], and highlight MDA-MB-231 cells as a valuable model system to study the ferroptosis-inducing mechanisms of these cytotoxic complexes. Ferroptosis induction by SCs is well documented, particularly in leukemia cells, and structure-activity relationship studies have revealed substantial differences in activity and ferroptosis selectivity by introducing halogens into the salophene ring [28]. Despite this promising anti-tumoral profile, the further development of SC-based ferroptosis inducers has been hindered by a limited understanding of the ferroptotic mechanisms. SCs increase mitochondrial ROS levels [28], indicating oxidative stress [79] that is associated with apoptosis [80], necroptosis [81], and ferroptosis [1]. Furthermore, their cytotoxic activity is attenuated by selective ferroptosis inhibitors and by increasing the intracellular antioxidant capacity through previously unknown mechanisms [28,29]. Our data indicate that SCs effectively catalyze peroxide-dependent redox reactions, apparently via a redox cycle involving the reduction of Fe(III) to Fe(II) and the reversible formation of oxo-bridged dimeric complexes, thereby generating reactive oxygen species, propagating the (per)oxidation of 20:4 and 22:4 in membrane PE and PI, and depleting cellular redox cofactors, i.e., NADPH (Fig. 6).

The development of ferroptosis inducers focuses on i) the suppression of GPX4 activity, either by inhibition, inactivation or degradation of the enzyme, ii) inhibition of system X_c_^−^, iii) depletion of the reduced form of CoQ10 (CoQ10H_2_) by targeting FSP1, iv) interference with the KEAP1-NRF2 axis, and v) induction of lipid peroxidation by organic peroxides, PUFA supplementation, or Fenton-active iron [2,24,25,82]. The latter includes Fe(II) complexes that, in the presence of H_2_O_2_, generate OH radicals that attack all types of biomolecules, including polyunsaturated membrane lipids, leading to membrane peroxidation and ultimately cell death by ferroptosis [83]. SCs have the basic requirements for such an activity: i) they switch between Fe(II) and Fe(III) states, ii) have an exchangeable axial ligand that allows oxygen or H_2_O_2_ access to the central ion, and iii) generate organic solvent radicals (methoxy radicals with methanol as vehicle) in the presence of H_2_O_2_, as observed for labile Fe(II). However, in contrast to Fe(II), SCs do not seem to generate OH radicals efficiently, as suggested by an OH radical-specific indicator reaction, while at the same time being markedly more potent in inducing membrane peroxidation. The identity of the primary radicals could not be determined. Likely candidates are hydroperoxide (OOH) radicals formed from Fe(III) and H_2_O_2_ by homolysis of the Fe–O bond of initially formed Fe(III)OOH complexes [84]. The latter have also been reported to react directly with organic molecules [84]. Whether the secondary methoxy radicals formed under our experimental conditions initiate membrane peroxidation has not been addressed but seems likely, given the substantially higher bond dissociation energy of methanol compared to the bis-allylic positions in PUFAs [85]. In addition, SCs are very effective in propagating lipid peroxidation, likely by accepting lipid peroxides and promoting the radical chain reaction that sustains progressive membrane peroxidation [86]. Note that SCs are not dependent on H_2_O_2_ to induce lipid peroxidation, as shown for artificial membranes under defined cell-free conditions. Whether the initial radicals triggering membrane peroxidation are generated from O_2_ or derive from traces of phospholipid hydroperoxides rapidly formed by autoxidation [87] remains elusive.

SCs undergo redox cycling that sustains lipid peroxidation in lipophilic membrane compartments, generate radicals to trigger membrane peroxidation, and are potent and efficient inducers of ferroptosis. Unlike free Fe(II), the complexed Fe(II/III) central atom in SCs is not subject to rapid oxidation and incorporation into ferritin, the Fenton-inactive storage form of iron [88]. Less well understood is why SCs outperform other ferroptosis inducers, such as RSL3, and why different cell death programs are induced depending on the cell line and tumor type studied [28,29,31,[33], [34], [35], [36], [37], [38],^40^,^41^] and depending on small changes in SC structure [[28], [29], [30]]. The interplay of multiple redox-active mechanisms appears to be the key to understanding these favorable properties of SCs. On the one hand, SCs catalyze DNA cleavage, leading to DNA single- and double-strand breaks [31,32,38,48,50,51]. Since apoptosis induction does not correlate with DNA cleavage activity in structure-activity relationship studies [32], the relevance of this mechanism for overall cell death induction is questionable. On the other hand, we show here, using the redox-sensitive sensor molecule TMB and the intracellular redox cofactor NADPH, that SCs are effective catalysts that utilize H_2_O_2_ for oxidation reactions, requiring a two-electron transfer for NADPH [89]. While we speculate that this catalytic oxidation is not limited to NADPH, but applies to multiple redox cofactors and antioxidants, the reaction is to some extent selective, considering that the GSH/GSSG ratio is maintained upon treatment with SCs.

NADPH is a biomarker for ferroptosis resistance in cancer cells [90,91] and its hydrolysis to NADH by the cytosolic phosphatase MESH1 determines ferroptosis sensitivity [92]. NAD(P)H also plays a central role in cellular antioxidant defense independently of its function in the FSP1/CoQ10 system [89]. By providing reducing equivalents, NADPH regenerates GSH from glutathione disulfide (GSSG) via glutathione reductase, reduces oxidized thioredoxin via thioredoxin reductase [89], and reactivates catalase that has been inactivated by H_2_O_2_ [93]. As a central redox cofactor for reductive anabolic reactions, NADPH is also involved in fatty acid [94], steroid [95], amino acid, and nucleotide biosynthesis [96] and the mevalonate pathway [95], all of which are interestingly linked to ferroptosis [1,7,[97], [98], [99], [100]], but also to other cell death programs, particularly apoptosis [[101], [102], [103]]. However, NADPH is a double-edged sword and, as a co-substrate of POR or NADPH oxidase isoenzymes, also contributes to lipid peroxidation and cell death under certain conditions [11,12,104]. Together, the competition between direct membrane peroxidation and the depletion of NADPH (and possibly other redox factors) may explain why SCs are potent ferroptosis inducers in cells susceptible to oxidative membrane damage, while favoring alternative cell death programs in less vulnerable cells.

## 5 Conclusion

SCs are anti-tumor redox catalysts with in vivo activity in preclinical studies. They induce massive lipid peroxidation, preferentially by incorporating three oxygens into 20:4 and 22:4 of membrane phospholipids associated with ferroptosis. As a consequence, SCs potently induce ferroptosis at nanomolar concentrations in a wide variety of cells, including therapy-resistant cell lines, and are particularly effective against mesenchymal, triple-negative breast cancer cells. The induction of cell death by SCs depends on redox cycling involving Fe(II) and Fe(III) and the reversible formation of μ-oxo dimers. As a result, SCs i) generate specific ROS that might initiate phospholipid hydroperoxide formation, ii) propagate membrane peroxidation, and iii) deplete cells of NADPH and potentially other redox cofactors and antioxidants that maintain redox homeostasis and counteract ferroptosis and other cell death pathways.

## Availability of data

The mass spectrometry lipidomics data (http://dx.doi.org/10.21228/M8423Z) have been deposited to Metabolomics Workbench: An international repository for metabolomics data and metadata, metabolite standards, protocols, tutorials and training, and analysis tools [105]. All other data are included in the manuscript or supplementary information or will be made available on request.

## CRediT authorship contribution statement

**Fengting Su:** Writing – review & editing, Writing – original draft, Visualization, Methodology, Investigation, Formal analysis, Data curation. **Hubert Descher:** Writing – review & editing, Visualization, Methodology, Investigation. **Minh Bui-Hoang:** Writing – review & editing, Visualization, Methodology, Investigation. **Hermann Stuppner:** Writing – review & editing, Supervision. **Ira Skvortsova:** Resources. **Ehsan Bonyadi Rad:** Resources, Funding acquisition. **Claudia Ascher:** Investigation. **Alexander Weiss:** Methodology, Supervision, Writing – review & editing. **Zhigang Rao:** Writing – review & editing, Supervision, Methodology. **Stephan Hohloch:** Writing – review & editing, Supervision, Resources, Methodology. **Solveigh C. Koeberle:** Writing – review & editing, Supervision, Methodology. **Ronald Gust:** Writing – review & editing, Supervision, Resources, Methodology. **Andreas Koeberle:** Writing – review & editing, Writing – original draft, Supervision, Project administration, Methodology, Funding acquisition, Data curation, Conceptualization.

## Declaration of Generative AI and AI-assisted technologies in the writing process

During the preparation of this work the authors used DeepL Write in order to improve readability and language. After using this tool, the authors reviewed and edited the content as needed and take full responsibility for the content of the publication.

## Declaration of competing interest

The authors declare that they have no known competing financial interests or personal relationships that could have appeared to influence the work reported in this paper.

## Data Availability

Data will be made available on request.
