# Supplementary material for: Iron(III)-salophene catalyzes redox cycles that induce phospholipid peroxidation and deplete cancer cells of ferroptosis-protecting cofactors
Source: Redox Biol. 2024 Jun 26;75:103257. doi: 10.1016/j.redox.2024.103257 (PMC11263665; doi:10.1016/j.redox.2024.103257)
Supplement: Multimedia component 1 [file mmc1.pdf]

Supplementary Material

Iron(III)-salophene catalyzes redox cycles that induce phospholipid peroxidation and deplete cancer cells of ferroptosis-protecting cofactors

Fengting Su[a], Hubert Descher[b], Minh Bui-Hoang[a,c], Hermann Stuppner[c], Ira Skvortsova[d], Ehsan Bonyadi Rad[a], Claudia Ascher[e], Alexander Weiss[e], Zhigang Rao[a], Stephan Hohloch[f], Solveigh C. Koeberle[a], Ronald Gust[b], Andreas Koeberle[a,]*

[a] Michael Popp Institute, Center for Molecular Biosciences Innsbruck (CMBI), University of Innsbruck, Innsbruck, Austria

[b] Institute of Pharmacy/Pharmaceutical Chemistry, Center for Molecular Biosciences Innsbruck (CMBI), University of Innsbruck, Innsbruck, Austria

[c] Unit of Pharmacognosy, Institute of Pharmacy, Center for Molecular Biosciences Innsbruck (CMBI), University of Innsbruck, Innsbruck, Austria

[d] EXTRO-Lab, Department of Therapeutic Radiology and Oncology, Medical University of Innsbruck, Innsbruck, Austria

[e] Institute for Biomedical Aging Research, University of Innsbruck, Innsbruck, Austria

[f] Institute for General, Inorganic and Theoretical Chemistry, University of Innsbruck, Innsbruck, Austria

*Correspondence to: Michael Popp Institute, Center for Molecular Biosciences Innsbruck (CMBI), University of Innsbruck, Mitterweg 24, 6020 Innsbruck, Austria; e-mail: andreas.koeberle@uibk.ac.at

Tables

Table S1. Mass spectrometric parameters for the analysis of phospholipids

Parameters	PC	PE	PI	oxPC[1]	oxPE[2]	oxPI[3]
Curtain gas	40 psi	40 psi	40 psi	40 psi	40 psi	40 psi
Collision gas pressure	medium	medium	medium	medium	medium	medium
Ion spray voltage	-4500 V	-4500 V	-4500 V	-4500 V	-4500 V	-4500 V
Heated capillary temperature	350°C	650°C	500°C	350°C	650°C	500°C
Sheath gas pressure	55 psi	55 psi	55 psi	55 psi	55 psi	55 psi
Auxiliary gas	75 psi	75 psi	75 psi	75 psi	75 psi	75 psi
Declustering potential	-44 V	-50 V	-44 V	-55 V (1[O])<br>-55 V (2[O])<br>-45 V (3[O])	-50 V	-50 V
Entrance potential	-10 V	-10 V	-10 V	-10 V	-10 V	-10 V
Collision energy	-46 eV	-38 eV	-62 eV	-52.5 eV (1[O])<br>-41.6 eV (2[O])<br>-43.2 eV (3[O])	-45 eV	-62 eV
Collision cell exit potential	-11 V	-12 V	-11 V	-23 V (1[O])<br>-24 V (2[O])<br>-20 V (3[O])	-12 V	-11 V

[1](per)oxidized PC; [2](per)oxidized PE; [3](per)oxidized PI

Table S2. Q3-fragment ions of oxidized 20:4 and 22:4

	20:4 Q3 (m/z)	22:4 Q3 (m/z)	Fragment ions
+3[O]	351.3	379.3	$[PUFA+3O-H]^-$
	349.3	377.3	$[PUFA+3O-2H]^-$
	347.3	375.3	$[PUFA+3O-4H]^-$
	333.3	361.3	$[PUFA+3O-H_2O-H]^-$
	331.3	359.3	$[PUFA+3O-H_2O-3H]^-$
	315.3	343.3	$[PUFA+3O-2H_2O-H]^-$
+2[O]	335.3	363.3	$[PUFA+2O-H]^-$
	331.3	359.3	$[PUFA+2O-5H]^-$
	317.3	345.3	$[PUFA+2O-H_2O-H]^-$
+1[O]	319.3	347.3	$[PUFA+O-H]^-$
	317.3	345.3	$[PUFA+O-3H]^-$
	301.3	329.3	$[PUFA+O-H_2O-H]^-$

Table S3. Q1 and Q3 masses and retention time for oxidized PC species

Species	Q1 (m/z)	Q3 (m/z)	Retention time (min)
PC(16:0_20:4+1[O])	856.6	255.2	3.01–4.6
PC(16:0_20:4+1[O])	856.6	319.2	3.01–4.6
PC(16:0_20:4+1[O])	856.6	301.2	3.32–3.5
PC(16:0_20:4+2[O])	872.6	255.2	2.3–3.55
PC(16:0_20:4+2[O])	872.6	317.2	2.85–3.3
PC(16:0_20:4+2[O])	872.6	331.2	2.3–2.45, 2.85–3.03
PC(16:0_20:4+2[O])	872.6	335.2	3.3–3.55
PC(16:0_20:4+3[O])	888.6	255.2	1.3–2.05
PC(16:0_20:4+3[O])	888.6	351.2	1.3–2.05
PC(16:0_20:4+3[O])	888.6	333.2	1.51.73
PC(18:0_20:4+1[O])	884.6	283.3	3.7–5.6
PC(18:0_20:4+1[O])	884.6	319.2	3.7–5.6
PC(18:0_20:4+1[O])	884.6	301.2	4.55–5.025
PC(18:0_20:4+2[O])	900.6	283.3	1.3–3.3
PC(18:0_20:4+2[O])	900.6	317.2	1.3–3.3
PC(18:0_20:4+2[O])	900.6	335.2	3.15–3.3
PC(18:0_20:4+3[O])	916.6	283.3	1.37–2.7
PC(18:0_20:4+3[O])	916.6	351.2	1.9–2.7
PC(18:0_20:4+3[O])	916.6	315.2	1.37–1.42
PC(18:1_20:4+1[O])	882.6	281.3	2.98–4.95
PC(18:1_20:4+1[O])	882.6	319.2	2.98–4.95
PC(18:1_20:4+1[O])	882.6	301.2	2.98–4.25
PC(18:1_20:4+2[O])	898.6	281.3	3–3.9
PC(18:1_20:4+2[O])	896.6	317.2	3–3.9
PC(18:1_20:4+3[O])	914.6	281.3	1.35–2.4
PC(18:1_20:4+3[O])	914.6	351.2	1.35–2.4
PC(16:0_22:4+1[O])	884.6	255.2	2–4.7
PC(16:0_22:4+1[O])	884.6	347.2	3.55–4.7
PC(16:0_22:4+1[O])	884.6	345.2	2.75–2.85
PC(16:0_22:4+1[O])	884.6	329.2	2–2.6, 3.3–3.8
PC(16:0_22:4+2[O])	900.6	255.2	3.4–3.9
PC(16:0_22:4+2[O])	900.6	344.2	3.4–3.9
PC(18:0_22:4+1[O])	912.6	283.2	2.2–5.05
PC(18:0_22:4+1[O])	912.6	329.2	2.2–2.45, 3.23–3.5, 3.7–4.2, 4.85–5.05

Table S4. Q1 and Q3 masses and retention time for oxidized PE species

Species	Q1 (m/z)	Q3 (m/z)	Retention time (min)
PE(16:0_20:4+1[O])	754.5	255.2	2.87–3.67
PE(16:0_20:4+1[O])	754.5	319.2	2.87–3.15, 3.27–3.67
PE(16:0_20:4+1[O])	754.5	317.2	2.87–3.15, 3.27–3.67
PE(18:0_20:4+1[O])	782.5	283.3	3.39–5.97
PE(18:0_20:4+1[O])	782.5	319.3	3.39–5.97
PE(18:0_20:4+1[O])	782.5	317.3	3.39–4.15, 4.35–4.5, 4.98–5.25
PE(18:0_20:4+1[O])	782.5	301.3	3.6–4.35, 4.5–5.25
PE(18:0_20:4+2[O])	798.5	283.3	3.29–3.57, 3.81–4.63, 4.76–5.32
PE(18:0_20:4+2[O])	798.5	317.3	3.29–3.57, 3.81–4.63, 4.76–5.32
PE(18:0_20:4+2[O])	798.5	331.3	4.76–4.91
PE(18:0_20:4+2[O])	798.5	335.3	3.29–3.57, 3.81–4.63, 4.91–5.32
PE(18:0_20:4+3[O])	814.5	283.3	1.47–2.91
PE(18:0_20:4+3[O])	814.5	351.3	1.47–2.91
PE(18:0_20:4+3[O])	814.5	333.3	2.065–2.43
PE(18:0_20:4+3[O])	814.5	315.3	1.47–1.86
PE(18:0_20:4+3[O])	814.5	349.3	1.47–1.86
PE(18:1_20:4+1[O])	780.5	281.3	2.9–4.39, 4.54–4.9
PE(18:1_20:4+1[O])	780.5	319.3	2.9–4.39, 4.54–4.9
PE(18:1_20:4+1[O])	780.5	317.3	2.9–3.25, 3.38–3.47, 3.56–3.75
PE(18:1_20:4+1[O])	780.5	301.3	2.9–3.25, 3.56–3.75
PE(18:1_20:4+2[O])	796.5	281.3	2.26–2.65, 2.85–3.22
PE(18:1_20:4+2[O])	796.5	317.3	2.85–3.22
PE(18:1_20:4+2[O])	796.5	331.3	2.26–2.65
PE(18:1_20:4+2[O])	796.5	335.3	2.26–2.51
PE(18:1_20:4+3[O])	812.5	281.3	1.44–2.193, 2.28–2.42
PE(18:1_20:4+3[O])	812.5	351.3	1.44–2.1,
PE(18:1_20:4+3[O])	812.5	347.3	1.44–1.84
PE(18:1_20:4+3[O])	812.5	349.3	1.44–1.69, 1.84–2.193, 2.28–2.42
PE(18:1_20:4+3[O])	812.5	331.3	1.84–2.1
PE(16:0_22:4+1[O])	782.5	255.2	3.89–4.25
PE(16:0_22:4+1[O])	782.5	345.3	3.89–4.25
PE(18:0_22:4+2[O])	826.6	283.2	4.67–5.05
PE(18:0_22:4+2[O])	826.6	345.3	4.67–5.05
PE(18:0_22:4+3[O])	842.6	283.2	2.26–2.9
PE(18:0_22:4+3[O])	842.6	379.3	2.26–2.9

Table S5. Q1 and Q3 masses and retention time for oxidized PI species

Species	Q1 (m/z)	Q3 (m/z)	Retention time (min)
PI(16:0_20:4+1[O])	873.5	255.2	0.8–1.8
PI(16:0_20:4+1[O])	873.5	319.2	0.8–1.8
PI(16:0_20:4+3[O])	905.5	255.2	0.3–2.13
PI(16:0_20:4+3[O])	905.5	351.2	0.3–1.4
PI(16:0_20:4+3[O])	905.5	315.2	0.3–2.13
PI(18:0_20:4+1[O])	901.5	283.3	1.2–2.7
PI(18:0_20:4+1[O])	901.5	319.3	1.2–2.7
PI(18:0_20:4+1[O])	901.5	301.3	1.2–1.49, 1.65–1.87
PI(18:0_20:4+2[O])	917.5	283.3	0.95–1.95
PI(18:0_20:4+2[O])	917.5	317.3	1.05–1.95
PI(18:0_20:4+2[O])	917.5	335.3	0.95–1.68
PI(18:0_20:4+3[O])	933.5	283.3	0.5–2.12
PI(18:0_20:4+3[O])	933.5	351.3	0.5–1.55
PI(18:0_20:4+3[O])	933.5	333.3	0.5–1.2, 1.8–2.12
PI(18:0_20:4+3[O])	933.5	315.3	0.5–1.55
PI(18:1_20:4+1[O])	899.5	281.2	1.0–2
PI(18:1_20:4+1[O])	899.5	319.3	1.0–1.2
PI(18:1_20:4+2[O])	915.5	281.2	0.65–1.5
PI(18:1_20:4+2[O])	915.5	317.3	0.65–1.5
PI(18:1_20:4+2[O])	915.5	335.3	0.65–1
PI(18:1_20:4+3[O])	931.5	281.2	0.5–1.2
PI(18:1_20:4+3[O])	931.5	351.3	0.5–0.98
PI(18:1_20:4+3[O])	931.5	315.3	0.5–0.98
PI(18:1_20:4+3[O])	931.5	349.3	0.73–0.98
PI(18:1_20:4+3[O])	931.5	331.3	0.5–1.2
PI(18:0_22:4+1[O])	929.6	283.2	1.45–2.6
PI(18:0_22:4+1[O])	929.6	347.3	1.45–2.6
PI(18:0_22:4+1[O])	929.6	329.3	1.45–2.1, 2.3–2.6
PI(18:0_22:4+2[O])	945.6	283.2	0.5–2.1
PI(18:0_22:4+2[O])	945.6	345.3	0.5–2.1
PI(18:0_22:4+2[O])	945.6	363.3	0.9–1.7
PI(18:0_22:4+3[O])	961.6	283.2	0.5–1.2
PI(18:0_22:4+3[O])	961.6	379.3	0.5–1.2
PI(18:0_22:4+3[O])	961.6	361.3	0.5–1.2
PI(18:0_22:4+3[O])	961.6	363.3	0.5–1.2

Table S6. Standard potentials ($E_{1/2}$) of SCs in DCM under oxygen and inert gas atmosphere

		monomer		dimer	
compound	conditions	reduction ($Fe^{III/II}$)	oxidation ($Fe^{II/III}$)	reduction ($Fe^{III/II}$)	oxidation ($Fe^{II/III}$)
**2**	Argon	-724 mV	-654 mV	-[1]	-
**2**	Oxygen	-715 mV	-	-1401 mV	-1317 mV
µ-oxo-**2**	Argon	-	-	-1369 mV	-1310 mV
**3**	Argon	-715 mV	-644 mV	-	-
**3**	Oxygen	-735 mV	-	-1404 mV	-
µ-oxo-**3**	Argon	-	-	-1390 mV	-1333 mV

[1]no signal

Table S7. Standard potentials ($E_{1/2}$) of SCs in DMSO under oxygen and inert gas atmosphere

compound	conditions	monomer reduction ($Fe^{III/II}$)	monomer oxidation ($Fe^{II/III}$)	dimer reduction ($Fe^{III/II}$)	dimer oxidation ($Fe^{II/III}$)
**2**	Argon	-732 mV	-664 mV	-[1]	-
**2**	Oxygen	-743 mV	-684 mV	-1399 mV	-1350 mV
μ-oxo-**2**	Argon	-731 mV	-678 mV	-1399 mV	-1345 mV
**3**	Argon	-730 mV	-661 mV	-	-
**3**	Oxygen	-742 mV	-682 mV	-1370 mV	-1325 mV
μ-oxo-**3**	Argon	-729 mV	-676 mV	-1370 mV	-1327 mV

[1]no signal

Supplementary Figures

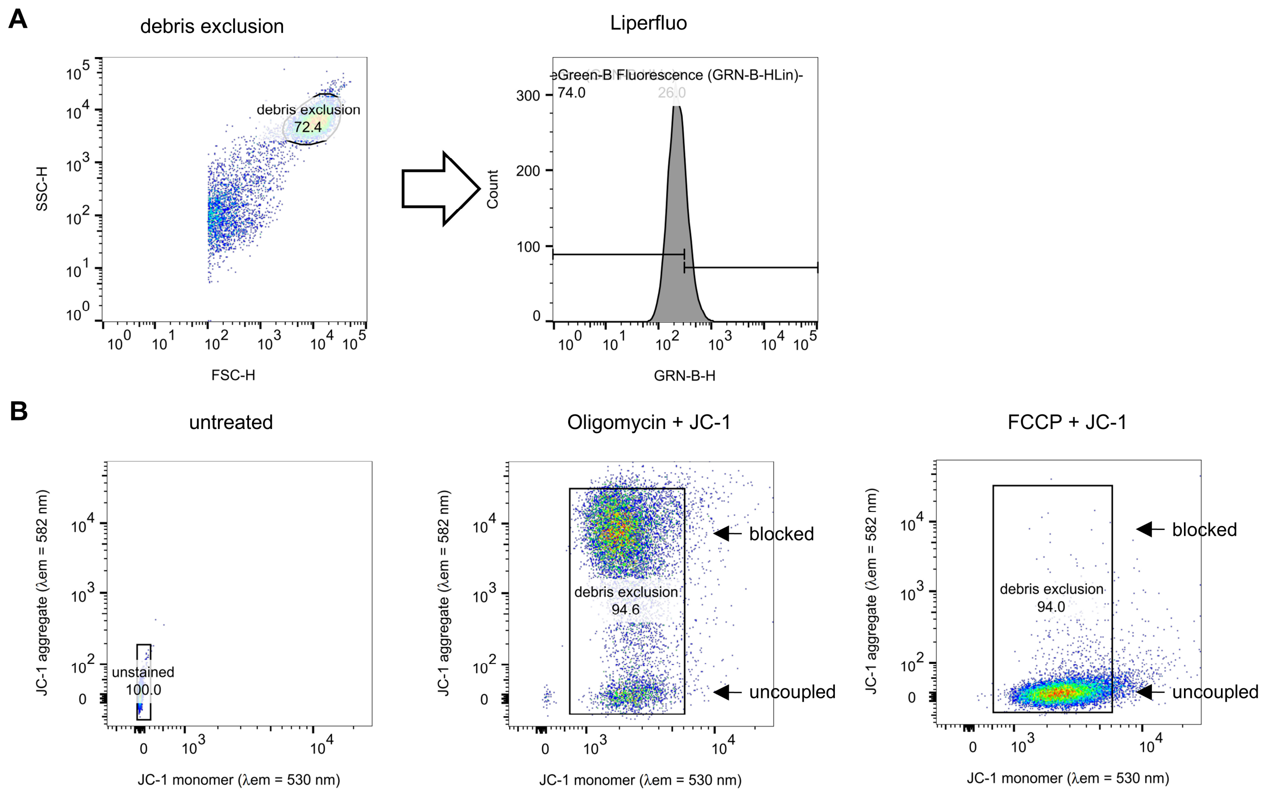

Supplementary Fig. 1. Gating strategy for flow cytometric analysis. A. Liperfluo staining of lipid peroxides. B. Determination of the mitochondrial membrane potential.

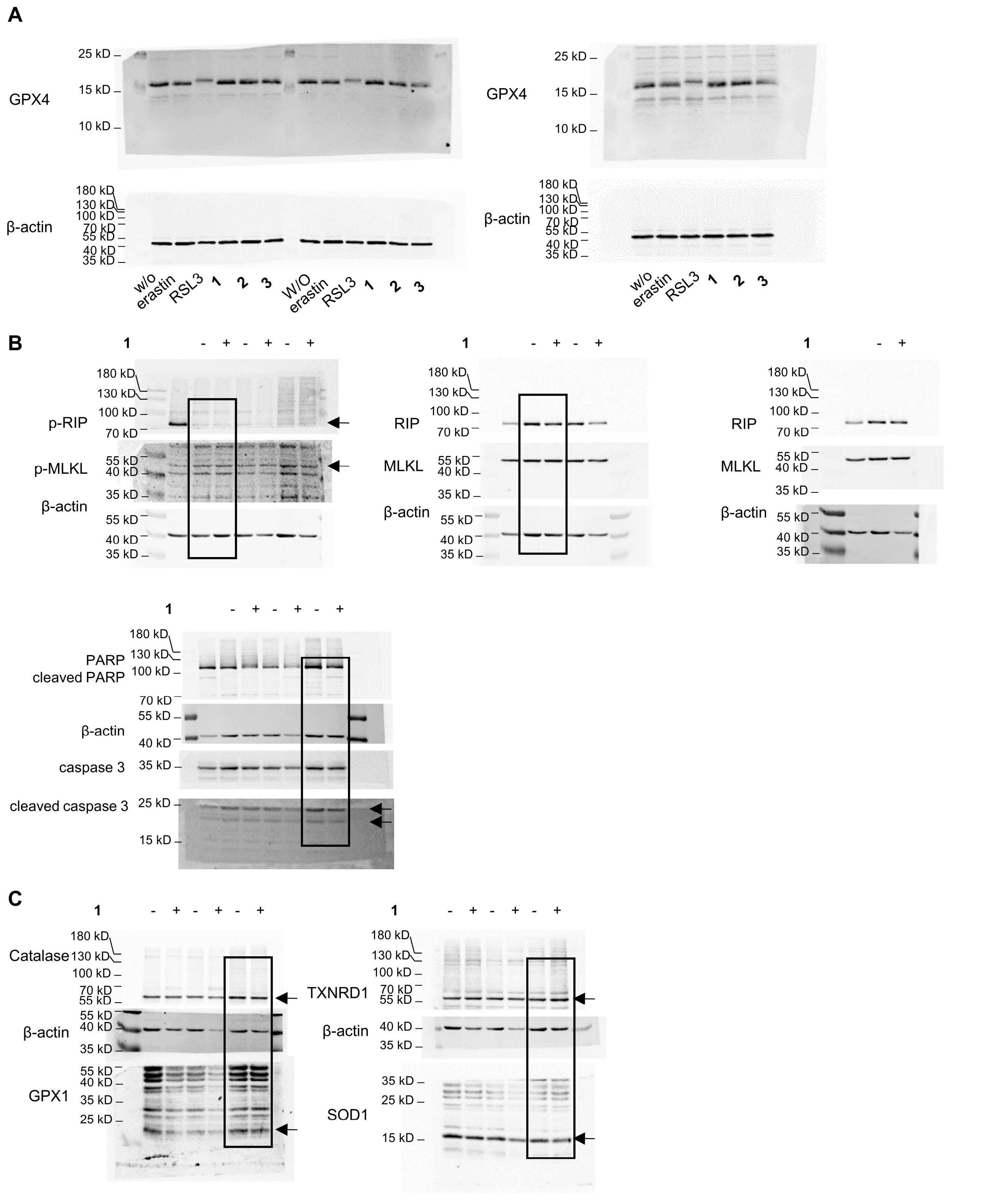

Supplementary Fig. 2. Uncropped versions of the blots presented in Fig. 4D (A), Supplementary Fig. 4B (B), and Supplementary Fig. 13A (C).

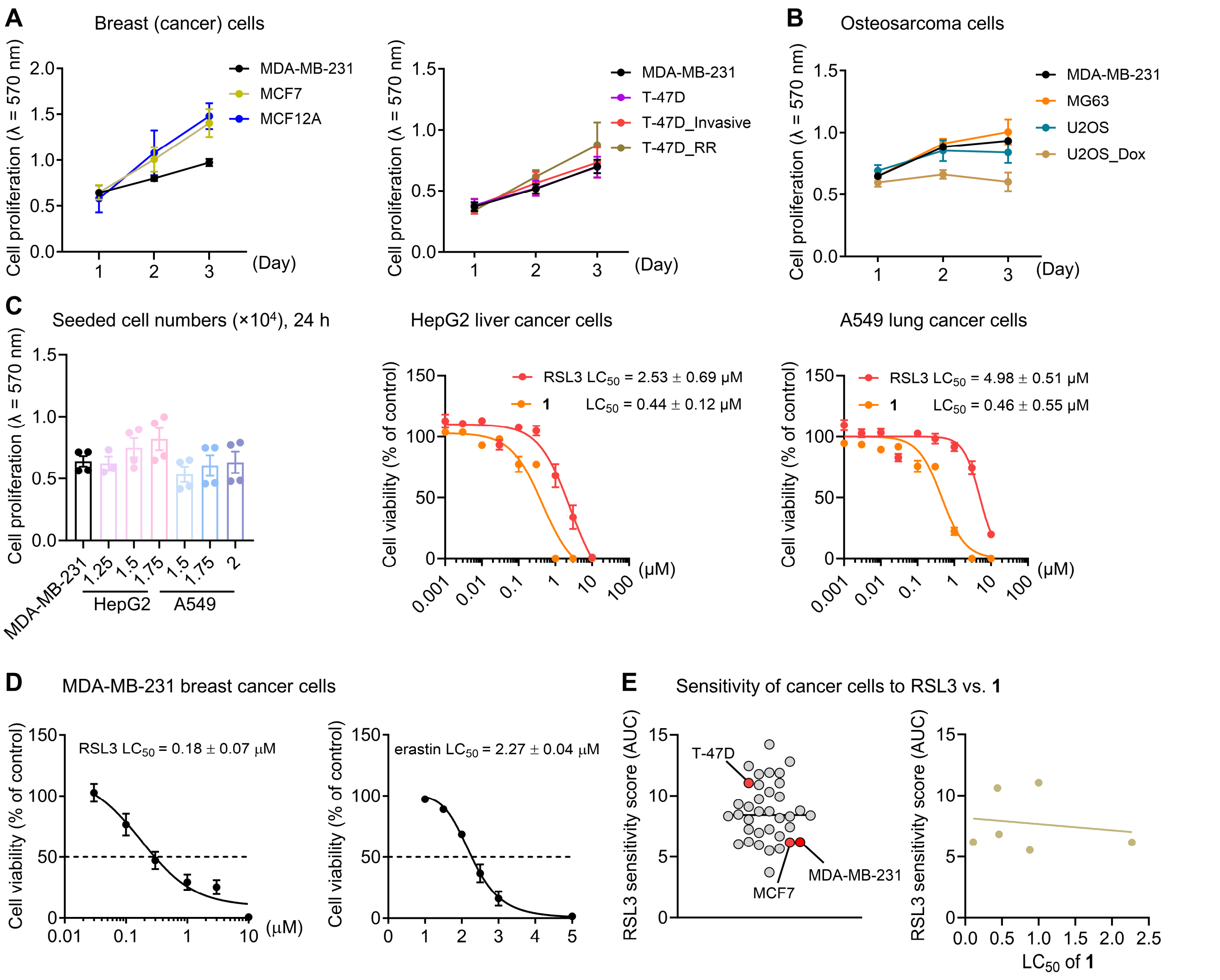

Supplementary Fig. 3. Proliferation rate of breast and osteosarcoma cell lines and ferroptosis sensitivity of cancer cell lines. A, B. Non-malignant breast cells, breast cancer cells (A) and osteosarcoma cells (B) were seeded in 96-well plates and incubated for 1-3 days. Cell proliferation / cell viability was assessed by measuring the cellular dehydrogenase activity (MTT assay). A. Non-malignant human breast cells (MCF12A: 1×10^4) and breast cancer cells (MDA-MB-231: 2×10^4; MCF7: 1×10^4, T-47D: 1.5×10^4, invasive T-47D cells, T-47D_invasive: 2×10^4, radioresistant T-47D cells, T-47D_RR: 7.5×10^3). B. Human osteosarcoma cells (U2OS: 1.5×10^4, MG63: 7.5×10^3, doxorubicin-resistant U2OS cells, U2OS_Dox: 1.5×10^4). C. The bar chart shows the proliferation of HepG2 and A549 cells within 24 h depending on the number of cells seeded in 96-well plates; MDA-MB-231 cells: 2×10^4. C, D. The line charts show the viability of cells seeded in 96-well plates and incubated (after 24 h) with vehicle (‘w/o’, 0.5% DMSO), **1** (0.001–10 µM; C), RSL3 (0.001–10 µM; C, D), or erastin (1-5 µM; D) for 48 h. LC_{50} values were calculated by non-linear regression analysis. C. Concentration-dependent effect on the viability of human HepG2 hepatocellular carcinoma (1.25×10^4) and human A549 lung carcinoma cells (2×10^4). D. Concentration-dependent effect on MDA-MB-231 (2×10^4) cell viability. E, RSL3 sensitivity scores for human breast cancer cell lines from the Cancer Therapeutics Response Portal (Broad Institute, https://portals.broadinstitute.org/ctrp/), expressed as area under the

curve (AUC; left panel). The dot plot correlates the LC_{50} values of **1** determined by MTT assay and RSL3 sensitivity scores for MDA-MB-231, MCF7, T-47D, MG63, HepG2, and A549 cells (right panel). Data are expressed as mean ± SEM from n = 3 (A, MCF7, MCF12A; T-47D_RR; B, C, D - erastin), n = 4 (A, T-47D_Invasive; D - RSL3), n = 5 (A - right panel, MDA-MB-231, T-47D), n = 6 (A - left panel, MDA-MB-231) independent experiments.

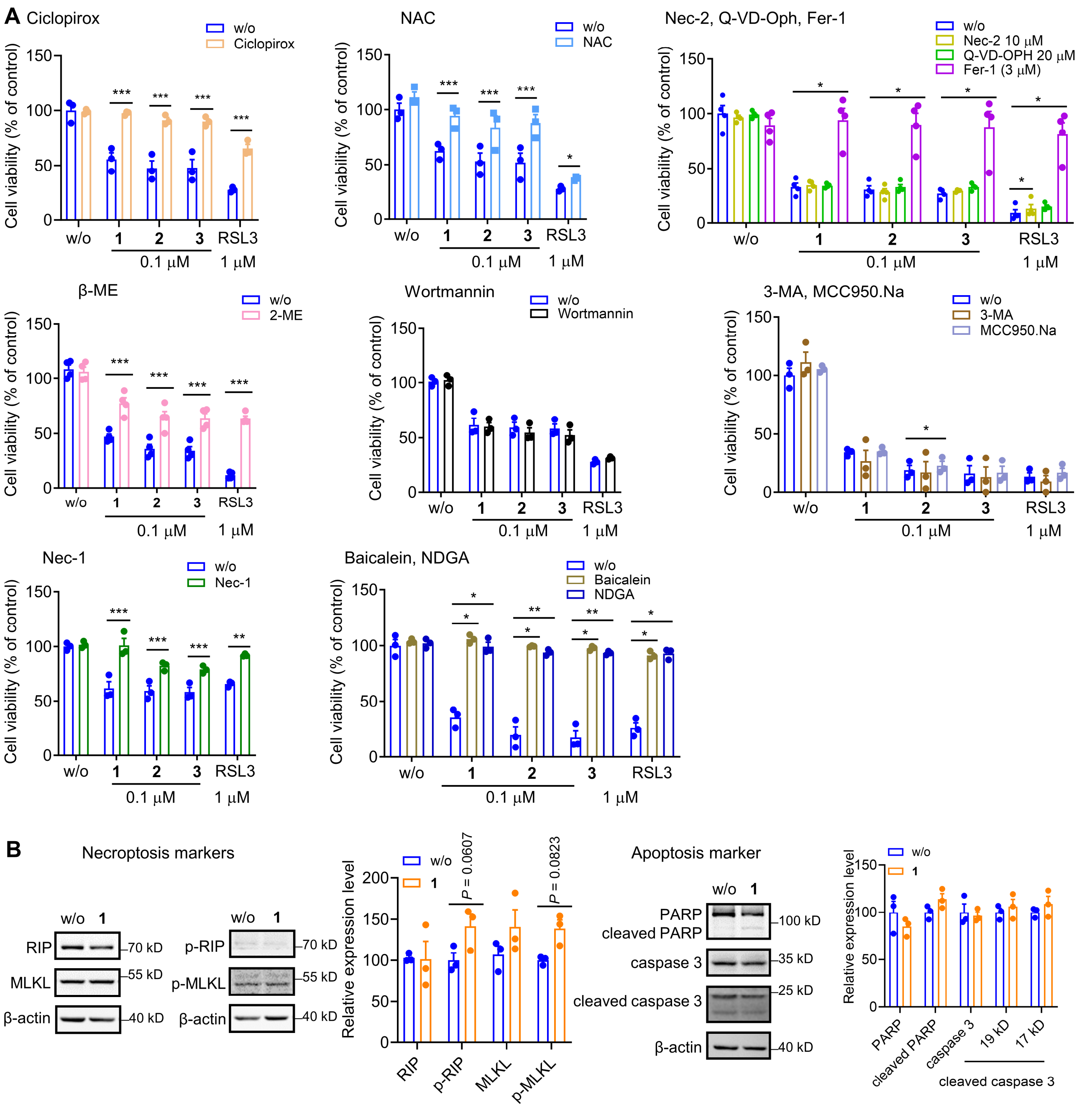

Supplementary Fig. 4. Cell death induction in triple negative breast cancer cells by SC is independent of lipoxygenases and preferentially relies on ferroptosis. A. MDA-MB-231 cells (2×10^4/well of a 96-well plate) were incubated with vehicle (‘w/o’, 0.5% DMSO), RSL3 (1 μM), **1**, **2**, or **3** (0.1 μM) for 48 h in the presence or the absence of cell death or redox-type LOX inhibitors before cell viability was determined. Ferroptosis inhibitors: Fer-1 (3 μM), ciclopirox (0.25 μM), N-acetyl-*L*-cysteine (NAC, 2.5 mM), β-mercaptoethanol (β-ME, 200 μM), necrostatin-1 (Nec-1, 40 μM; also inhibits necroptosis); necroptosis inhibitor: necrostatin-2 (Nec-2, 10 μM); apoptosis inhibitor: Q-VD-OPH (20 μM); autophagy inhibitors: wortmannin (1 μM), 3-methyladenine (3-MA, 1 mM); pyroptosis inhibitor: MCC950.Na (1 μM); redox-type LOX inhibitors: pan-LOX inhibitors baicalein (3 μM) and NDGA (3 μM). B. Phosphorylation and expression of apoptosis and necroptosis markers in MDA-MB-231 cells

(5×10^5/well of a 6-well plate) incubated (after 24 h) with vehicle (‘w/o’, 0.1% DMSO) or **1** (0.3 μM) for 24 h. Data are expressed as mean ± SEM from n = 3 (A, ciclopirox, wortmannin, 3-MA, MCC950.Na, Nec-1, Baicalein, NDGA, Fer-1 (lower right), BLX3887, CAY10698, CJ-13610; B), n = 4 (A, NEC-2, Q-VD-OPH, Fer-1 (middle left)), n = 6 (A, NAC, β-ME) independent experiments. $^{*}P < 0.05$, $^{**}P < 0.01$, $^{***}P < 0.001$ for comparisons indicated by bars; repeated measures two-way ANOVA + Sidak's *post hoc* tests (A, ciclopirox, NAC, β-ME, Wortmanin, Nec-1; B) or Dunnett's *post hoc* tests (A, Nec-2, Q-VD-OPH, Fer-1, 3-MA, MCC950.Na, Baicalein, NDGA).

0 100 1000
% of w/o

		w/o	RSL3	RSL3 + Fer-1	**1**	**2**	**3**
PE oxidation	PE(16:0_20:4 + 1[O])	0.06 ± 0.13	0.23 ± 0.06	0.04 ± 0.06	0.29 ± 0.09	0.26 ± 0.06	0.41 ± 0.13
	PE(18:0_20:4 + 1[O])	2.15 ± 2.18	4.97 ± 0.94	1.89 ± 1.14	5.95 ± 1.52	5.04 ± 1.14	7.28 ± 2.29
	PE(18:1_20:4 + 1[O])	0.39 ± 0.79	1.32 ± 0.27	0.3 ± 0.38	1.68 ± 0.48	1.46 ± 0.24	2.08 ± 0.62
	PE(16:0_22:4 + 1[O])	0.24 ± 0.4	0.89 ± 0.22	0.15 ± 0.24	1.15 ± 0.36	0.94 ± 0.11	1.31 ± 0.39
	PE(18:0_20:4 + 2[O])	0.15 ± 0.4	0.4 ± 0.07	0.11 ± 0.11	0.72 ± 0.22	0.61 ± 0.26	0.92 ± 0.39
	PE(18:1_20:4 + 2[O])	0.05 ± 0.13	0.16 ± 0.03	0.03 ± 0.04	0.21 ± 0.06	0.18 ± 0.07	0.24 ± 0.11
	PE(18:0_22:4 + 2[O])	0.01 ± 0.03	0.03 ± 0.01	0.01 ± 0	0.05 ± 0.01	0.05 ± 0.01	0.07 ± 0.03
	PE(18:0_20:4 + 3[O])	0.06 ± 0.7	0.77 ± 0.23	0.03 ± 0.26	1.49 ± 0.58	1.14 ± 0.5	1.27 ± 0.49
	PE(18:1_20:4 + 3[O])	0.03 ± 0.48	0.35 ± 0.11	0.01 ± 0.12	0.75 ± 0.29	0.58 ± 0.32	0.69 ± 0.4
	PE(18:0_22:4 + 3[O])	0 ± 0.03	0.07 ± 0.03	0 ± 0.02	0.11 ± 0.04	0.09 ± 0.02	0.1 ± 0.01
PC oxidation	PC(16:0_20:4 + 1[O])	0.12 ± 0.01	0.2 ± 0.01	0.1 ± 0.01	0.41 ± 0.03	0.32 ± 0.03	0.43 ± 0.08
	PC(18:0_20:4 + 1[O])	0.16 ± 0.02	0.33 ± 0.02	0.14 ± 0.04	0.59 ± 0.07	0.49 ± 0.06	0.52 ± 0.07
	PC(18:1_20:4 + 1[O])	0.19 ± 0.03	0.34 ± 0.01	0.16 ± 0.02	0.64 ± 0.08	0.52 ± 0.05	0.63 ± 0.1
	PC(16:0_22:4 + 1[O])	0.29 ± 0.01	0.3 ± 0.02	0.26 ± 0.03	0.24 ± 0.02	0.21 ± 0.01	0.34 ± 0.06
	PC(18:0_22:4 + 1[O])	0.9 ± 0.12	0.88 ± 0.1	0.91 ± 0.15	0.53 ± 0.03	0.46 ± 0.06	0.78 ± 0.2
	PC(16:0_20:4 + 2[O])	0.07 ± 0	0.09 ± 0.01	0.06 ± 0.01	0.19 ± 0.01	0.18 ± 0.01	0.22 ± 0.07
	PC(18:0_20:4 + 2[O])	0.02 ± 0	0.03 ± 0	0.01 ± 0	0.09 ± 0.01	0.09 ± 0	0.09 ± 0.02
	PC(18:1_20:4 + 2[O])	0.02 ± 0	0.06 ± 0.01	0.01 ± 0	0.28 ± 0.02	0.29 ± 0.01	0.33 ± 0.11
	PC(16:0_22:4 + 2[O])	0.002 ± 0	0 ± 0	0 ± 0	0.03 ± 0.01	0.03 ± 0	0.03 ± 0.01
	PC(16:0_20:4 + 3[O])	0 ± 0	0.03 ± 0.01	0 ± 0	0.2 ± 0.04	0.16 ± 0.03	0.17 ± 0.03
	PC(18:0_20:4 + 3[O])	0 ± 0	0.03 ± 0	0 ± 0	0.21 ± 0.03	0.16 ± 0.05	0.16 ± 0.03
	PC(18:1_20:4 + 3[O])	0 ± 0	0.02 ± 0	0 ± 0	0.18 ± 0.02	0.13 ± 0.02	0.13 ± 0.02
PI oxidation	PI(16:0_20:4 + 1[O])	0.01 ± 0	0.03 ± 0	0.01 ± 0	0.05 ± 0	0.05 ± 0	0.07 ± 0.01
	PI(18:0_20:4 + 1[O])	0.37 ± 0.06	1.18 ± 0.13	0.33 ± 0.06	2.07 ± 0.14	1.77 ± 0.11	2.55 ± 0.76
	PI(18:1_20:4 + 1[O])	0.02 ± 0.01	0.08 ± 0.01	0.02 ± 0	0.19 ± 0.02	0.17 ± 0.01	0.27 ± 0.08
	PI(18:0_22:4 + 1[O])	0.07 ± 0.01	0.2 ± 0.02	0.07 ± 0.01	0.25 ± 0.01	0.22 ± 0.02	0.3 ± 0.09
	PI(18:0_20:4 + 2[O])	0.04 ± 0.01	0.15 ± 0.02	0.03 ± 0	0.57 ± 0.06	0.53 ± 0.03	0.66 ± 0.2
	PI(18:1_20:4 + 2[O])	0 ± 0	0.02 ± 0.01	0 ± 0	0.07 ± 0.01	0.07 ± 0	0.1 ± 0.03
	PI(18:0_22:4 + 2[O])	0.02 ± 0	0.05 ± 0.01	0.02 ± 0	0.15 ± 0.02	0.13 ± 0.01	0.18 ± 0.06
	PI(16:0_20:4 + 3[O])	0.03 ± 0	0.05 ± 0.01	0.03 ± 0	0.09 ± 0.01	0.07 ± 0.01	0.1 ± 0.03
	PI(18:0_20:4 + 3[O])	0.06 ± 0.01	0.67 ± 0.11	0.04 ± 0	2.54 ± 0.5	2.01 ± 0.26	2.4 ± 0.73
	PI(18:1_20:4 + 3[O])	0.03 ± 0.01	0.13 ± 0.02	0.01 ± 0.01	0.67 ± 0.09	0.57 ± 0.07	0.7 ± 0.25
	PI(18:0_22:4 + 3[O])	0.01 ± 0.01	0.14 ± 0.02	0.01 ± 0	0.3 ± 0.06	0.26 ± 0.03	0.31 ± 0.11

Supplementary Fig. 5. SCs efficiently induce phospholipid peroxidation in triple negative breast cancer cells. MDA-MB-231 cells (3.12×10^6/75 cm^2) were treated with vehicle (‘w/o’, 0.1% DMSO), RSL3 (1 µM), RSL3 together with Fer-1 (3 µM), **1**, **2**, or **3** (1 µM each) for 2 h, and oxidized PE, PC, and PI species were analyzed by UPLC-MS/MS. Numbers indicate absolute amounts in pmol per 1×10^6 cells and the color expresses percentage changes relative to vehicle. Data are expressed as mean ± SEM from n = 3 independent experiments.

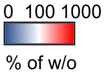

		PE(18:0_20:4) 1[O]	2[O]	3[O]
	w/o	1.34 ± 0.05	0.11 ± 0	0.1 ± 0.01
	RSL3	2.5 ± 0.08	0.27 ± 0.01	0.52 ± 0.06
	Erastin	1.8 ± 0.09	0.12 ± 0.01	0.15 ± 0.02
**1** (µM)	0.001	1.52 ± 0.15	0.11 ± 0.02	0.11 ± 0.03
	0.01	1.64 ± 0.03	0.13 ± 0.01	0.12 ± 0.02
	0.1	2.87 ± 0.43	0.32 ± 0.06	0.58 ± 0.15
	1	3.8 ± 0.76	0.65 ± 0.01	1.39 ± 0.1
	10	4.32 ± 0.39	0.81 ± 0.13	2.32 ± 0.54
**2** (µM)	0.001	1.84 ± 0.13	0.15 ± 0.02	0.14 ± 0.01
	0.01	1.99 ± 0.26	0.18 ± 0.01	0.21 ± 0.05
	0.1	2.94 ± 0.26	0.36 ± 0.01	0.7 ± 0.04
	1	3.82 ± 0.13	0.61 ± 0.08	1.36 ± 0.23
	10	4.84 ± 0.61	0.94 ± 0.15	2.46 ± 0.55
**3** (µM)	0.001	1.64 ± 0.16	0.15 ± 0.01	0.13 ± 0.02
	0.01	1.96 ± 0.28	0.18 ± 0.03	0.21 ± 0.04
	0.1	2.82 ± 0.33	0.32 ± 0.04	0.58 ± 0.12
	1	3.76 ± 0.29	0.47 ± 0.04	0.92 ± 0.12
	10	4.51 ± 0.46	0.81 ± 0.14	2 ± 0.41

Supplementary Fig. 6. Concentration-dependent oxidation of PE(18:0_20:4) by SCs. MDA-MB-231 cells (3.12×10^6/75 cm^2) were treated with vehicle (‘w/o’, 0.1% DMSO), RSL3 (1 µM), erastin (2 µM), **1**, **2**, or **3** (0.001–10 µM each) for 2 h, and oxidized PE, PC, and PI species were analyzed by UPLC-MS/MS. Numbers indicate absolute amounts in pmol per 1×10^6 cells and the color expresses percentage changes relative to vehicle. Data are expressed as mean ± SEM from n = 3 independent experiments.

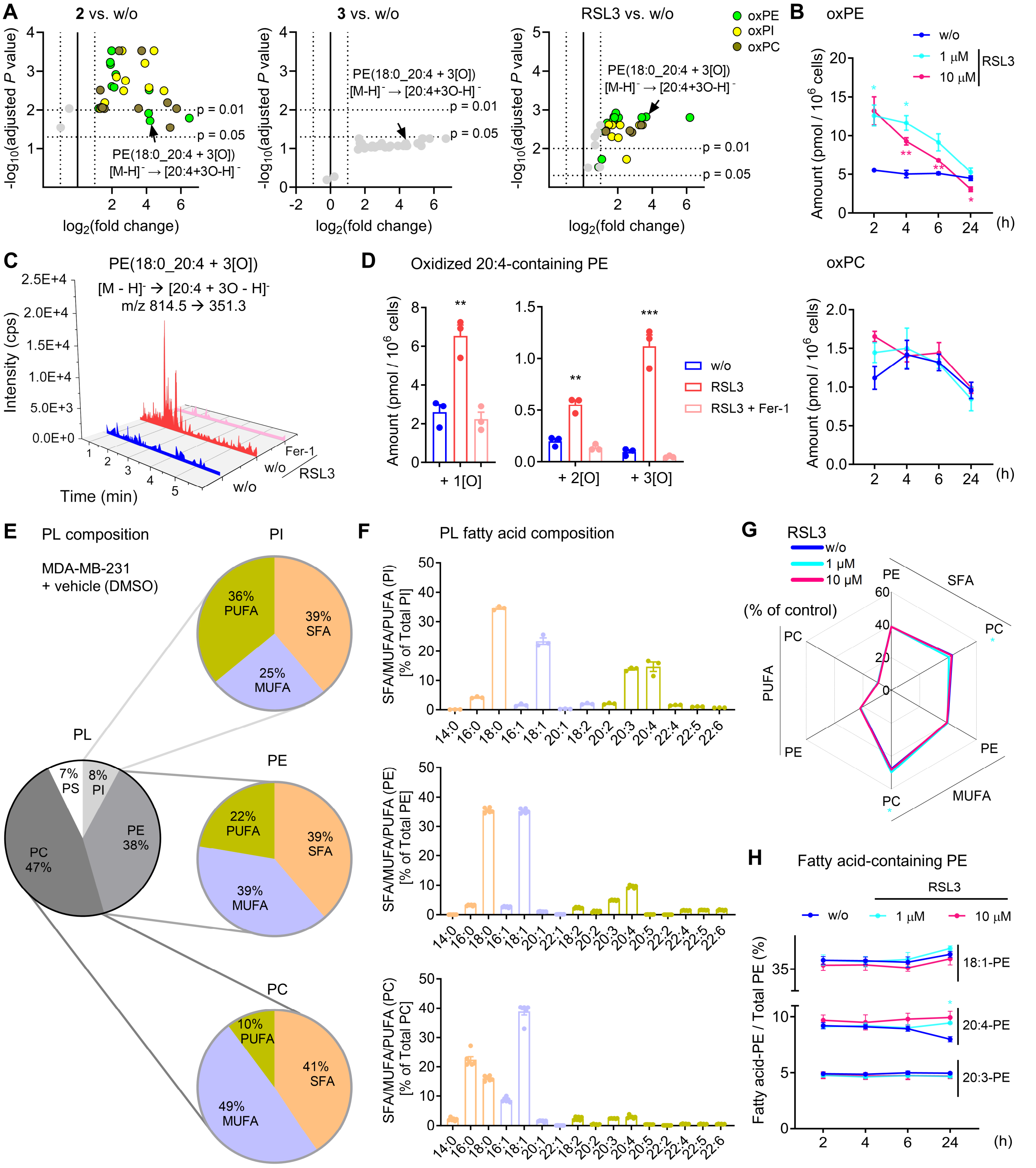

Supplementary Fig. 7. Composition of the (redox) phospholipidome of triple negative breast cancer cells challenged with RSL3 or SCs. MDA-MB-231 cells (3.12×10^6/75 cm^2) were treated with vehicle (‘w/o’, 0.1% DMSO), **2**, **3** (1 µM each), RSL3 (1 µM unless otherwise noted), or RSL3 plus Fer-1 (3 µM) for 2 h (unless otherwise noted), and oxidized and non-oxidized PE, PC, and PI species were analyzed by UPLC-MS/MS. A. Volcano plots showing the log2 of fold-change in the amount of (per)oxidized PC, PE, and PI species relative to vehicle control and the negative log10(adjusted *P* value) calculated vs. vehicle control; two-tailed multiple unpaired Student t tests with correction for

multiple comparisons (false discovery rate 5%). B. Time- and concentration-dependent changes in the cellular levels of total (per)oxidized PE and PC. C. Extracted chromatograms based on the fragmentation of $[PE(18:0\_20:4+3[O]-H]^-$ to $[20:4+3[O]-H]^-$. D. Amount of PE carrying 20:4 with 1[O], 2[O], or [3O] incorporated. E. Relative phospholipid composition of vehicle-treated MDA-MB-231 cells and the proportions of SFAs, MUFAs, and PUFAs in phospholipid subclasses. F. Proportions of individual fatty acids in major phospholipid classes. G. RSL3 does not substantially affect the proportion of SFAs, MUFAs, or PUFAs in PC or PE. H. Concentration-dependent effect of RSL3 on selected PE species. Data are expressed as mean ± SEM from n = 3 (PI), n = 6 (PE, PC) independent experiments. $^{*}P < 0.05$, $^{**}P < 0.01$, $^{***}P < 0.001$ compared to vehicle control; repeated measures two-way ANOVA + Dunnett's (B, D right panel, G, H) or repeated measures one-way ANOVA + Dunnett's *post hoc* tests (D left panel).

0 100 1000
% of w/o

	RSL3 (µM)	2 h w/o	2 h 1	2 h 10	4 h w/o	4 h 1	4 h 10	6 h w/o	6 h 1	6 h 10	24 h w/o	24 h 1	24 h 10
PE oxidation	PE(16:0_20:4 +1[O])	0.06 ± 0	0.23 ± 0.04	0.19 ± 0.03	0.07 ± 0.01	0.23 ± 0.02	0.16 ± 0.02	0.07 ± 0	0.15 ± 0.02	0.11 ± 0.01	0.05 ± 0	0.08 ± 0.01	0.05 ± 0
	PE(18:0_20:4 +1[O])	2.53 ± 0.13	4.89 ± 0.69	5.24 ± 0.75	2.28 ± 0.29	4.74 ± 0.47	3.71 ± 0.23	2.38 ± 0.13	3.98 ± 0.57	3.01 ± 0.07	2.09 ± 0.21	2.44 ± 0.21	1.48 ± 0.16
	PE(18:1_20:4 +1[O])	0.95 ± 0.12	1.76 ± 0.28	1.76 ± 0.34	0.96 ± 0.05	1.52 ± 0.15	1.31 ± 0.07	0.92 ± 0.04	1.25 ± 0.18	0.96 ± 0.04	0.67 ± 0.07	0.78 ± 0.1	0.43 ± 0.04
	PE(16:0_22:4 +1[O])	0.04 ± 0.01	0.09 ± 0.02	0.09 ± 0.03	0.05 ± 0	0.08 ± 0.01	0.08 ± 0.02	0.04 ± 0	0.06 ± 0.01	0.04 ± 0.01	0.02 ± 0	0.03 ± 0	0.02 ± 0
	PE(18:0_22:4 +1[O])	0.57 ± 0.05	0.86 ± 0.1	0.79 ± 0.12	0.54 ± 0.09	0.85 ± 0.09	0.54 ± 0.03	0.51 ± 0.03	0.67 ± 0.07	0.44 ± 0.02	0.59 ± 0.01	0.45 ± 0.01	0.23 ± 0.03
	PE(16:0_20:4 +2[O])	0.06 ± 0	0.08 ± 0.01	0.08 ± 0	0.05 ± 0.01	0.09 ± 0.01	0.07 ± 0	0.05 ± 0	0.07 ± 0.01	0.05 ± 0.01	0.04 ± 0.01	0.06 ± 0	0.06 ± 0.01
	PE(18:0_20:4 +2[O])	0.51 ± 0.05	1.07 ± 0.1	1.12 ± 0.17	0.39 ± 0.01	1.05 ± 0.08	0.89 ± 0.04	0.43 ± 0.03	0.84 ± 0.09	0.65 ± 0.01	0.38 ± 0.02	0.48 ± 0.06	0.32 ± 0.03
	PE(18:1_20:4 +2[O])	0.2 ± 0.02	0.42 ± 0.01	0.41 ± 0.05	0.19 ± 0.03	0.33 ± 0.05	0.27 ± 0.04	0.19 ± 0.02	0.24 ± 0.03	0.2 ± 0.02	0.18 ± 0.01	0.18 ± 0.02	0.12 ± 0.01
	PE(16:0_22:4 +2[O])	0.04 ± 0.01	0.07 ± 0.01	0.06 ± 0.01	0.03 ± 0	0.06 ± 0.01	0.05 ± 0	0.03 ± 0	0.06 ± 0	0.04 ± 0	0.02 ± 0	0.03 ± 0.01	0.03 ± 0.01
	PE(18:0_22:4 +2[O])	0.02 ± 0.01	0.11 ± 0.02	0.12 ± 0.03	0.01 ± 0	0.13 ± 0.01	0.09 ± 0.01	0.02 ± 0	0.09 ± 0.01	0.07 ± 0.01	0.01 ± 0	0.05 ± 0.01	0.03 ± 0.01
	PE(16:0_20:4 +3[O])	0.02 ± 0.01	0.23 ± 0.01	0.23 ± 0.02	0.02 ± 0	0.18 ± 0.02	0.13 ± 0.01	0.01 ± 0	0.11 ± 0.01	0.07 ± 0.01	0.02 ± 0	0.03 ± 0.01	0.01 ± 0
	PE(18:0_20:4 +3[O])	0.25 ± 0.05	1.55 ± 0.05	1.73 ± 0.2	0.21 ± 0.02	1.32 ± 0.11	1.06 ± 0.05	0.2 ± 0.04	0.9 ± 0.04	0.64 ± 0.01	0.17 ± 0.02	0.32 ± 0.06	0.13 ± 0.02
	PE(18:1_20:4 +3[O])	0.2 ± 0.04	0.89 ± 0.05	0.99 ± 0.09	0.17 ± 0.01	0.7 ± 0.04	0.61 ± 0.04	0.2 ± 0.02	0.47 ± 0.06	0.36 ± 0.03	0.19 ± 0.02	0.22 ± 0.05	0.12 ± 0.02
	PE(16:0_22:4 +3[O])	0.02 ± 0	0.11 ± 0.02	0.15 ± 0.02	0.01 ± 0	0.12 ± 0.01	0.1 ± 0.01	0.01 ± 0	0.09 ± 0.02	0.06 ± 0	0.02 ± 0	0.04 ± 0.01	0.02 ± 0.01
	PE(18:0_22:4 +3[O])	0.04 ± 0	0.25 ± 0.02	0.26 ± 0.03	0.04 ± 0	0.27 ± 0.02	0.19 ± 0.02	0.03 ± 0.01	0.16 ± 0.01	0.11 ± 0	0.04 ± 0	0.06 ± 0.01	0.02 ± 0
PC oxidation	PC(16:0_20:4 +1[O])	0.16 ± 0.02	0.24 ± 0.01	0.22 ± 0.05	0.16 ± 0.01	0.26 ± 0.03	0.16 ± 0.02	0.16 ± 0.01	0.2 ± 0.02	0.16 ± 0.02	0.1 ± 0.01	0.12 ± 0.01	0.09 ± 0.02
	PC(18:0_20:4 +1[O])	0.14 ± 0.01	0.24 ± 0.02	0.26 ± 0.03	0.14 ± 0.02	0.27 ± 0.03	0.21 ± 0.03	0.15 ± 0.01	0.22 ± 0.02	0.2 ± 0.03	0.13 ± 0.02	0.15 ± 0.01	0.12 ± 0.01
	PC(18:1_20:4 +1[O])	0.15 ± 0.02	0.24 ± 0.01	0.24 ± 0.02	0.13 ± 0.02	0.26 ± 0.03	0.21 ± 0.02	0.15 ± 0.01	0.21 ± 0.03	0.17 ± 0.02	0.08 ± 0.01	0.14 ± 0.02	0.09 ± 0.01
	PC(16:0_22:4 +1[O])	0.09 ± 0.01	0.11 ± 0.01	0.1 ± 0.02	0.1 ± 0.01	0.11 ± 0.01	0.1 ± 0.01	0.09 ± 0.01	0.09 ± 0.01	0.12 ± 0.02	0.05 ± 0	0.08 ± 0.01	0.11 ± 0.02
	PC(18:0_22:4 +1[O])	0.21 ± 0.02	0.22 ± 0.03	0.16 ± 0.03	0.19 ± 0.03	0.18 ± 0.02	0.16 ± 0.02	0.17 ± 0.01	0.15 ± 0.03	0.12 ± 0.02	0.07 ± 0.01	0.11 ± 0.01	0.09 ± 0.01
	PC(16:0_20:4 +2[O])	0.2 ± 0.03	0.23 ± 0.02	0.12 ± 0.02	0.2 ± 0.02	0.19 ± 0.03	0.12 ± 0.02	0.19 ± 0.03	0.2 ± 0.01	0.11 ± 0.03	0.08 ± 0.01	0.11 ± 0	0.11 ± 0.01
	PC(18:0_20:4 +2[O])	0.04 ± 0	0.04 ± 0	0.04 ± 0.01	0.04 ± 0	0.03 ± 0.01	0.05 ± 0	0.04 ± 0	0.04 ± 0	0.03 ± 0.01	0.02 ± 0	0.03 ± 0.01	0.03 ± 0.01
	PC(18:1_20:4 +2[O])	0.06 ± 0	0.07 ± 0.01	0.06 ± 0.01	0.05 ± 0	0.04 ± 0.01	0.05 ± 0.01	0.05 ± 0.01	0.05 ± 0	0.04 ± 0.01	0.02 ± 0	0.02 ± 0	0.03 ± 0
	PC(16:0_20:4 +3[O])	0.14 ± 0.02	0.14 ± 0.01	0.14 ± 0	0.12 ± 0.03	0.11 ± 0.01	0.12 ± 0.03	0.11 ± 0.02	0.1 ± 0.02	0.09 ± 0.02	0.08 ± 0.02	0.1 ± 0.01	0.11 ± 0.02
	PC(18:0_20:4 +3[O])	0.06 ± 0	0.06 ± 0.01	0.05 ± 0.01	0.06 ± 0.01	0.06 ± 0.01	0.05 ± 0.01	0.07 ± 0.01	0.06 ± 0.01	0.06 ± 0.01	0.03 ± 0	0.05 ± 0.01	0.08 ± 0
	PC(18:1_20:4 +3[O])	0.11 ± 0.01	0.1 ± 0.01	0.08 ± 0.01	0.09 ± 0.01	0.11 ± 0.02	0.09 ± 0.02	0.1 ± 0.01	0.11 ± 0.02	0.09 ± 0	0.06 ± 0.01	0.1 ± 0.01	0.1 ± 0.02

Supplementary Fig. 8. Time- and concentration-dependent oxidation of PE and PC species in triple negative breast cancer cells challenged with RSL3. MDA-MB-231 cells (3.12×10^6/75 cm^2) were treated with vehicle ('w/o', 0.1% DMSO) or RSL3, and oxidized PE and PC species were analyzed by UPLC-MS/MS. Numbers indicate absolute amounts in pmol per 1×10^6 cells and the color expresses percentage changes relative to vehicle. Data are expressed as mean ± SEM from n = 3 independent experiments.

50 100 150

% of w/o

PE species	w/o	RSL3	RSL3 + Fer-1	**1** (µM)			**2** (µM)			**3** (µM)		
				1	3	10	1	3	10	1	3	10
14:0_16:0	0.03 ± 0	0.03 ± 0	0.03 ± 0	0.03 ± 0	0.03 ± 0	0.04 ± 0	0.03 ± 0	0.04 ± 0	0.03 ± 0	0.03 ± 0	0.03 ± 0	0.04 ± 0
16:0_16:0	0.06 ± 0	0.05 ± 0	0.06 ± 0	0.07 ± 0	0.07 ± 0.01	0.08 ± 0	0.07 ± 0	0.08 ± 0	0.08 ± 0	0.07 ± 0	0.07 ± 0	0.08 ± 0.01
16:0_18:0	0.7 ± 0.04	0.65 ± 0.02	0.73 ± 0.02	0.86 ± 0.07	0.96 ± 0.12	1.15 ± 0.09	0.94 ± 0.08	1.03 ± 0.09	1.08 ± 0.07	0.86 ± 0.06	0.95 ± 0.06	0.99 ± 0.08
18:0_18:0	0.15 ± 0.02	0.13 ± 0.02	0.17 ± 0.01	0.17 ± 0.01	0.17 ± 0.03	0.18 ± 0.02	0.18 ± 0.02	0.16 ± 0.02	0.16 ± 0.01	0.17 ± 0.01	0.16 ± 0.01	0.15 ± 0.02
16:0_16:1	0.46 ± 0.04	0.43 ± 0.05	0.45 ± 0.05	0.41 ± 0.04	0.42 ± 0.02	0.42 ± 0.02	0.4 ± 0.03	0.4 ± 0.01	0.43 ± 0.02	0.41 ± 0.03	0.41 ± 0.03	0.42 ± 0.02
18:0_16:1	3.12 ± 0.24	3.09 ± 0.05	3.14 ± 0.19	3.2 ± 0.14	3.28 ± 0.13	3.35 ± 0.19	3.23 ± 0.18	3.18 ± 0.2	3.2 ± 0.16	2.94 ± 0.17	3 ± 0.12	3.06 ± 0.19
14:0_18:1	0.07 ± 0	0.06 ± 0	0.07 ± 0	0.07 ± 0	0.07 ± 0	0.08 ± 0	0.07 ± 0	0.07 ± 0	0.07 ± 0	0.07 ± 0	0.07 ± 0	0.07 ± 0
16:0_18:1	3.26 ± 0.16	3.07 ± 0.1	3.18 ± 0.14	3.3 ± 0.11	3.42 ± 0.12	3.78 ± 0.13	3.42 ± 0.14	3.44 ± 0.12	3.6 ± 0.16	3.18 ± 0.14	3.2 ± 0.03	3.46 ± 0.07
16:1_18:1	1.31 ± 0.11	1.21 ± 0.12	1.27 ± 0.11	1.15 ± 0.11	1.15 ± 0.1	1.11 ± 0.06	1.15 ± 0.1	1.1 ± 0.09	1.16 ± 0.09	1.18 ± 0.1	1.17 ± 0.08	1.19 ± 0.08
18:0_18:1	29.81 ± 1.42	30.64 ± 0.7	29.51 ± 1.12	32.46 ± 1.53	33.45 ± 1.78	35.17 ± 1.91	32.57 ± 2.11	34.52 ± 1.03	34.3 ± 1.71	31.88 ± 1.54	32.1 ± 1.81	33.68 ± 1.68
18:1_18:1	12.64 ± 0.59	12.38 ± 0.2	13.03 ± 0.41	13.03 ± 0.34	13.3 ± 0.64	13.87 ± 0.73	12.91 ± 0.66	13.26 ± 0.55	13.44 ± 0.6	13 ± 0.68	13.23 ± 0.74	13.77 ± 0.81
18:1_20:1	2.06 ± 0.03	2.19 ± 0.06	2.06 ± 0.05	2.17 ± 0.09	2.22 ± 0.07	2.32 ± 0.12	2.28 ± 0.1	2.39 ± 0.13	2.31 ± 0.09	2.29 ± 0.02	2.32 ± 0.07	2.44 ± 0.06
16:1_22:1	0.24 ± 0.01	0.25 ± 0.02	0.23 ± 0.01	0.26 ± 0.01	0.25 ± 0.01	0.24 ± 0.01	0.25 ± 0.01	0.25 ± 0.02	0.25 ± 0.01	0.25 ± 0.01	0.26 ± 0.02	0.26 ± 0.01
16:0_18:2	0.21 ± 0.01	0.19 ± 0.02	0.21 ± 0.02	0.18 ± 0.01	0.17 ± 0.01	0.18 ± 0.01	0.18 ± 0.01	0.18 ± 0.01	0.19 ± 0.01	0.18 ± 0.01	0.18 ± 0.01	0.18 ± 0.01
18:0_18:2	3.08 ± 0.11	2.99 ± 0.07	3.2 ± 0.17	3.16 ± 0.09	3.08 ± 0.1	3.15 ± 0.18	3.04 ± 0.14	2.98 ± 0.14	2.99 ± 0.13	2.92 ± 0.12	3.03 ± 0.14	2.97 ± 0.12
18:1_18:2	1.56 ± 0.12	1.43 ± 0.14	1.61 ± 0.09	1.37 ± 0.14	1.38 ± 0.09	1.33 ± 0.07	1.41 ± 0.15	1.31 ± 0.12	1.38 ± 0.12	1.47 ± 0.14	1.53 ± 0.12	1.5 ± 0.14
18:0_20:2	2.57 ± 0.05	2.53 ± 0.1	2.52 ± 0.06	2.68 ± 0.12	2.61 ± 0.09	2.62 ± 0.11	2.69 ± 0.06	2.64 ± 0.1	2.63 ± 0.09	2.65 ± 0.07	2.63 ± 0.1	2.66 ± 0.09
16:0_20:3_ω6	0.11 ± 0	0.11 ± 0.01	0.11 ± 0	0.09 ± 0.01	0.09 ± 0	0.09 ± 0	0.09 ± 0.01	0.08 ± 0	0.09 ± 0	0.09 ± 0.01	0.09 ± 0	0.09 ± 0
16:0_20:3_ω9	0.13 ± 0.01	0.11 ± 0.01	0.11 ± 0.01	0.11 ± 0.01	0.1 ± 0.01	0.1 ± 0	0.1 ± 0	0.1 ± 0	0.09 ± 0.01	0.11 ± 0.01	0.1 ± 0.01	0.1 ± 0.01
16:1_20:3_ω6	0.02 ± 0	0.02 ± 0	0.01 ± 0	0.01 ± 0	0.01 ± 0	0.01 ± 0	0.01 ± 0	0.01 ± 0	0.01 ± 0	0.01 ± 0	0.02 ± 0	0.01 ± 0
16:1_20:3_ω9	0.03 ± 0	0.03 ± 0	0.02 ± 0	0.02 ± 0	0.02 ± 0	0.02 ± 0	0.02 ± 0	0.02 ± 0	0.02 ± 0	0.02 ± 0	0.02 ± 0	0.02 ± 0
18:0_20:3_ω6	3.47 ± 0.08	3.43 ± 0.12	3.45 ± 0.13	3.29 ± 0.12	3.2 ± 0.16	3.19 ± 0.1	3.2 ± 0.13	3.17 ± 0.1	3.21 ± 0.09	3.34 ± 0.04	3.31 ± 0.06	3.23 ± 0.05
18:0_20:3_ω9	4.55 ± 0.25	4.55 ± 0.39	4.67 ± 0.17	4.63 ± 0.07	4.52 ± 0.08	4.28 ± 0.18	4.56 ± 0.17	4.5 ± 0.34	4.31 ± 0.12	4.68 ± 0.1	4.75 ± 0.21	4.55 ± 0.26
18:1_20:3_ω6	0.43 ± 0.03	0.42 ± 0.05	0.43 ± 0.02	0.37 ± 0.04	0.34 ± 0.02	0.32 ± 0.02	0.36 ± 0.03	0.34 ± 0.03	0.35 ± 0.02	0.4 ± 0.03	0.39 ± 0.02	0.37 ± 0.02
18:1_20:3_ω9	0.93 ± 0.05	0.89 ± 0.01	0.94 ± 0.04	0.84 ± 0.01	0.8 ± 0.01	0.69 ± 0.02	0.84 ± 0.03	0.79 ± 0.02	0.76 ± 0.03	0.83 ± 0.03	0.83 ± 0.02	0.77 ± 0.03
16:0_20:4	0.84 ± 0.07	0.78 ± 0.07	0.83 ± 0.08	0.6 ± 0.07	0.53 ± 0.04	0.43 ± 0.02	0.58 ± 0.07	0.48 ± 0.04	0.5 ± 0.03	0.62 ± 0.03	0.56 ± 0.05	0.53 ± 0.04
16:1_20:4	0.07 ± 0	0.09 ± 0.01	0.07 ± 0	0.06 ± 0	0.06 ± 0	0.05 ± 0	0.07 ± 0	0.06 ± 0	0.06 ± 0	0.07 ± 0.01	0.06 ± 0	0.07 ± 0
18:0_20:4	15.99 ± 0.1	15.92 ± 0.6	15.94 ± 0.4	15.23 ± 0.5	14.92 ± 0.59	13.55 ± 0.78	15.19 ± 0.37	14.39 ± 0.48	14.24 ± 0.44	15.64 ± 0.07	15.42 ± 0.61	14.31 ± 0.41
18:1_20:4	2.82 ± 0.21	2.8 ± 0.24	2.74 ± 0.2	2.04 ± 0.24	1.74 ± 0.1	1.47 ± 0.07	2.05 ± 0.25	1.69 ± 0.12	1.74 ± 0.11	2.3 ± 0.13	2.15 ± 0.14	1.92 ± 0.13
18:1_20:5_ω3	0.19 ± 0.02	0.17 ± 0.01	0.18 ± 0.01	0.13 ± 0.02	0.11 ± 0.01	0.09 ± 0	0.13 ± 0.02	0.1 ± 0.01	0.11 ± 0.01	0.15 ± 0.01	0.13 ± 0.01	0.12 ± 0.01
18:1_20:5_ω6	0.02 ± 0	0.02 ± 0	0.02 ± 0	0.02 ± 0	0.02 ± 0	0.01 ± 0	0.02 ± 0	0.02 ± 0	0.02 ± 0	0.02 ± 0	0.01 ± 0	0.02 ± 0
16:0_22:2	0.02 ± 0	0.02 ± 0	0.02 ± 0	0.03 ± 0	0.03 ± 0	0.03 ± 0	0.03 ± 0	0.03 ± 0	0.03 ± 0	0.03 ± 0	0.03 ± 0	0.03 ± 0
16:0_22:4	0.11 ± 0.01	0.11 ± 0.01	0.1 ± 0.01	0.1 ± 0.01	0.09 ± 0.01	0.09 ± 0.01	0.1 ± 0.01	0.1 ± 0.01	0.09 ± 0.01	0.09 ± 0.01	0.1 ± 0.01	0.1 ± 0.01
16:1_22:4	0.01 ± 0	0.02 ± 0	0.01 ± 0	0.02 ± 0	0.02 ± 0	0.02 ± 0	0.02 ± 0	0.02 ± 0	0.02 ± 0	0.01 ± 0	0.02 ± 0	0.02 ± 0
18:0_22:4	2.24 ± 0.09	2.55 ± 0.15	2.2 ± 0.15	2.14 ± 0.14	2.01 ± 0.16	1.93 ± 0.11	2.2 ± 0.16	2.11 ± 0.11	2.1 ± 0.08	2.21 ± 0.16	2.1 ± 0.17	1.94 ± 0.12
18:1_22:4	0.49 ± 0.02	0.56 ± 0.04	0.48 ± 0.04	0.47 ± 0.02	0.48 ± 0.04	0.42 ± 0.02	0.5 ± 0.04	0.47 ± 0.02	0.48 ± 0.02	0.5 ± 0.02	0.5 ± 0.03	0.48 ± 0.02
16:0_22:5_ω3	0.15 ± 0.01	0.15 ± 0.01	0.16 ± 0	0.11 ± 0	0.11 ± 0	0.09 ± 0	0.12 ± 0.01	0.1 ± 0.01	0.1 ± 0	0.13 ± 0	0.12 ± 0	0.1 ± 0.01
16:0_22:5_ω6	0.02 ± 0	0.01 ± 0	0.02 ± 0	0.01 ± 0	0.01 ± 0	0.01 ± 0	0.01 ± 0	0.01 ± 0	0.01 ± 0	0.01 ± 0	0.01 ± 0	0.01 ± 0
16:1_22:5_ω3	0.01 ± 0	0.01 ± 0	0.01 ± 0	0.01 ± 0	0.01 ± 0	0.01 ± 0	0.01 ± 0	0.01 ± 0	0.01 ± 0	0.01 ± 0	0.01 ± 0	0.01 ± 0
16:1_22:5_ω6	0.01 ± 0	0.01 ± 0	0.01 ± 0	0.01 ± 0	0.01 ± 0	0.01 ± 0	0.01 ± 0	0.01 ± 0	0.01 ± 0	0.01 ± 0	0.01 ± 0	0.01 ± 0
18:0_22:5_ω3	2.16 ± 0.02	2.29 ± 0.07	2.27 ± 0.11	2.04 ± 0.04	1.91 ± 0.09	1.64 ± 0.07	1.97 ± 0.09	1.81 ± 0.03	1.73 ± 0.07	2.02 ± 0.08	2 ± 0.13	1.79 ± 0.07
18:0_22:5_ω6	0.32 ± 0.02	0.33 ± 0	0.31 ± 0.01	0.3 ± 0	0.29 ± 0.01	0.27 ± 0.01	0.29 ± 0.01	0.27 ± 0.01	0.26 ± 0.01	0.3 ± 0.01	0.29 ± 0.01	0.27 ± 0
18:1_22:5_ω3	0.35 ± 0.01	0.35 ± 0.03	0.33 ± 0.02	0.26 ± 0.03	0.24 ± 0.01	0.19 ± 0.01	0.26 ± 0.03	0.23 ± 0.01	0.22 ± 0.02	0.28 ± 0.02	0.28 ± 0.01	0.25 ± 0.01
18:1_22:5_ω6	0.08 ± 0	0.09 ± 0	0.07 ± 0	0.07 ± 0	0.08 ± 0	0.07 ± 0	0.08 ± 0	0.07 ± 0	0.08 ± 0	0.08 ± 0.01	0.08 ± 0	0.08 ± 0
18:2_22:5_ω3	0.03 ± 0	0.02 ± 0	0.03 ± 0	0.02 ± 0	0.02 ± 0	0.01 ± 0	0.02 ± 0	0.02 ± 0	0.02 ± 0	0.02 ± 0	0.02 ± 0	0.02 ± 0
18:2_22:5_ω6	0.02 ± 0	0.01 ± 0	0.01 ± 0	0.01 ± 0	0.01 ± 0	0.01 ± 0	0.01 ± 0	0.01 ± 0	0.01 ± 0	0.01 ± 0	0.01 ± 0	0.01 ± 0
16:0_22:6	0.15 ± 0	0.13 ± 0.01	0.16 ± 0	0.12 ± 0	0.1 ± 0	0.08 ± 0	0.11 ± 0.01	0.09 ± 0.01	0.09 ± 0	0.12 ± 0.01	0.11 ± 0	0.09 ± 0
18:0_22:6	2.37 ± 0.21	2.16 ± 0.15	2.25 ± 0.16	1.87 ± 0.19	1.73 ± 0.19	1.49 ± 0.15	1.84 ± 0.2	1.52 ± 0.08	1.59 ± 0.15	1.85 ± 0.1	1.68 ± 0.15	1.41 ± 0.12
18:1_22:6	0.57 ± 0.06	0.54 ± 0.04	0.56 ± 0.05	0.39 ± 0.06	0.34 ± 0.03	0.27 ± 0.02	0.4 ± 0.07	0.32 ± 0.03	0.33 ± 0.03	0.46 ± 0.03	0.43 ± 0.03	0.35 ± 0.03

Supplementary Fig. 9. Short-term effect of SCs on the PE fatty acid composition of triple-negative breast cancer cells. MDA-MB-231 cells ($3.12\times10^6/75\ cm^2$) were treated with vehicle (‘w/o’, 0.1% DMSO), RSL3 (1 µM), RSL3 plus Fer-1 (3 µM), **1**, **2**, or **3** (1–10 µM) , and non-oxidized PE species were analyzed by UPLC-MS/MS. Numbers indicate the proportion of individual PE species relative to total PE (100%) and the color expresses percentage changes relative to vehicle. Data are expressed as mean ± SEM from n = 3 independent experiments.

50 100 150

% of w/o

PC species	w/o	RSL3	RSL3 + Fer-1	**1** (µM) 1	**1** (µM) 3	**1** (µM) 10	**2** (µM) 1	**2** (µM) 3	**2** (µM) 10	**3** (µM) 1	**3** (µM) 3	**3** (µM) 10
14:0_16:0	2.12 ± 0.13	1.94 ± 0.15	2.21 ± 0.14	1.98 ± 0.12	1.96 ± 0.09	1.96 ± 0.12	2.03 ± 0.16	1.91 ± 0.1	2 ± 0.13	2.08 ± 0.15	2.01 ± 0.16	1.9 ± 0.11
16:0_16:0	2.91 ± 0.08	2.67 ± 0.11	3.03 ± 0.01	3.06 ± 0.13	2.77 ± 0.18	3.16 ± 0.18	3 ± 0.18	2.86 ± 0.1	3.1 ± 0.09	3 ± 0.01	2.99 ± 0.07	3.17 ± 0.02
14:0_16:1	0.47 ± 0.02	0.47 ± 0	0.49 ± 0.01	0.44 ± 0.02	0.44 ± 0.01	0.41 ± 0.01	0.43 ± 0.02	0.4 ± 0.02	0.42 ± 0.02	0.44 ± 0.02	0.43 ± 0.01	0.41 ± 0.02
14:0_18:1	1.6 ± 0.11	1.55 ± 0.07	1.63 ± 0.09	1.57 ± 0.08	1.55 ± 0.09	1.45 ± 0.06	1.58 ± 0.12	1.5 ± 0.09	1.47 ± 0.07	1.63 ± 0.12	1.61 ± 0.09	1.52 ± 0.09
16:0_16:1	8.66 ± 0.44	8.47 ± 0.34	8.83 ± 0.49	8.37 ± 0.39	8.46 ± 0.45	8.49 ± 0.53	8.49 ± 0.32	8.16 ± 0.34	8.6 ± 0.47	8.47 ± 0.47	8.46 ± 0.36	8.34 ± 0.42
16:1_16:1	0.65 ± 0.03	0.63 ± 0	0.66 ± 0.02	0.62 ± 0.03	0.61 ± 0.03	0.59 ± 0.02	0.62 ± 0.04	0.59 ± 0.04	0.63 ± 0.03	0.64 ± 0.03	0.63 ± 0.02	0.61 ± 0.03
16:0_18:1	19.01 ± 0.3	18.96 ± 0.41	19.17 ± 0.57	20.29 ± 0.71	20.86 ± 0.72	21.92 ± 0.35	20.69 ± 0.61	21.78 ± 0.52	21.55 ± 0.58	20.34 ± 0.5	20.71 ± 0.4	21.54 ± 0.34
18:0_16:1	1.75 ± 0.03	1.79 ± 0.03	1.79 ± 0.02	1.92 ± 0.06	1.94 ± 0.03	2.05 ± 0.05	1.88 ± 0.06	1.98 ± 0.02	1.94 ± 0.05	1.82 ± 0	1.84 ± 0	1.87 ± 0.02
16:1_18:1	4.63 ± 0.08	4.66 ± 0.08	4.75 ± 0.09	4.64 ± 0.18	4.69 ± 0.15	4.57 ± 0.06	4.65 ± 0.19	4.61 ± 0.17	4.66 ± 0.11	4.79 ± 0.14	4.77 ± 0.13	4.65 ± 0.1
18:0_18:1	20.5 ± 0.47	20.97 ± 0.39	20.14 ± 0.47	21.86 ± 0.27	22.28 ± 0.64	22.97 ± 0.55	21.84 ± 0.86	22.23 ± 1	22.58 ± 0.24	21.33 ± 0.2	21.71 ± 0.11	22.46 ± 0.22
18:1_18:1	12.3 ± 0.3	12.51 ± 0.22	12.25 ± 0.27	13.52 ± 0.12	13.57 ± 0.21	13.37 ± 0.4	13.08 ± 0.06	13.74 ± 0.17	12.91 ± 0.08	13.01 ± 0.17	13.35 ± 0.29	13.14 ± 0.18
18:1_20:1	3.13 ± 0.1	3.18 ± 0.04	3.17 ± 0.1	3.31 ± 0.04	3.42 ± 0.11	3.44 ± 0.04	3.35 ± 0.04	3.53 ± 0.07	3.26 ± 0.03	3.21 ± 0.07	3.39 ± 0	3.44 ± 0.04
16:1_22:1	0.15 ± 0	0.15 ± 0	0.15 ± 0	0.16 ± 0	0.16 ± 0.01	0.16 ± 0.01	0.15 ± 0	0.16 ± 0.01	0.16 ± 0.01	0.15 ± 0	0.15 ± 0	0.16 ± 0.01
14:0_18:2	0.09 ± 0	0.1 ± 0	0.1 ± 0	0.09 ± 0	0.09 ± 0	0.08 ± 0	0.09 ± 0	0.08 ± 0	0.08 ± 0	0.09 ± 0	0.09 ± 0	0.08 ± 0
16:0_18:2	1.93 ± 0.02	1.92 ± 0.04	1.95 ± 0.07	1.82 ± 0.04	1.85 ± 0.03	1.72 ± 0.03	1.81 ± 0.02	1.72 ± 0.01	1.8 ± 0.05	1.81 ± 0.02	1.85 ± 0.06	1.72 ± 0.01
18:0_18:2	1.7 ± 0.01	1.69 ± 0.05	1.71 ± 0.04	1.7 ± 0.11	1.69 ± 0.06	1.62 ± 0.06	1.65 ± 0.13	1.68 ± 0.1	1.59 ± 0.11	1.57 ± 0.02	1.58 ± 0.02	1.59 ± 0.02
16:0_20:3_ω6	0.76 ± 0.03	0.74 ± 0.05	0.7 ± 0.04	0.64 ± 0	0.64 ± 0.02	0.57 ± 0.03	0.63 ± 0.03	0.6 ± 0.02	0.62 ± 0.01	0.67 ± 0.01	0.65 ± 0	0.62 ± 0.01
16:0_20:3_ω9	0.29 ± 0.02	0.28 ± 0.01	0.27 ± 0.02	0.26 ± 0.02	0.26 ± 0.02	0.23 ± 0.02	0.27 ± 0.02	0.25 ± 0.02	0.26 ± 0.02	0.28 ± 0.01	0.29 ± 0.01	0.27 ± 0
18:1_18:2	2.14 ± 0.08	2.07 ± 0.05	2.12 ± 0.08	1.94 ± 0.06	1.89 ± 0.03	1.73 ± 0.02	1.92 ± 0.03	1.76 ± 0.03	1.76 ± 0.04	1.89 ± 0.03	1.83 ± 0.04	1.7 ± 0.03
16:0_20:4	1.8 ± 0.18	1.76 ± 0.23	1.71 ± 0.16	1.29 ± 0.11	1.18 ± 0.07	0.93 ± 0.1	1.37 ± 0.12	1.15 ± 0.09	1.16 ± 0.08	1.53 ± 0.08	1.36 ± 0.04	1.22 ± 0.06
16:1_20:4	0.19 ± 0.03	0.19 ± 0.03	0.18 ± 0.02	0.13 ± 0.02	0.12 ± 0.01	0.11 ± 0.01	0.14 ± 0.02	0.12 ± 0.02	0.12 ± 0.01	0.16 ± 0.01	0.15 ± 0.01	0.13 ± 0.01
16:1_20:3_ω6	0.11 ± 0.01	0.11 ± 0.01	0.11 ± 0.01	0.09 ± 0.01	0.08 ± 0.01	0.07 ± 0.01	0.09 ± 0.01	0.08 ± 0.01	0.08 ± 0	0.09 ± 0	0.09 ± 0	0.08 ± 0
16:1_20:3_ω9	0.05 ± 0.01	0.05 ± 0.01	0.05 ± 0	0.05 ± 0	0.05 ± 0	0.04 ± 0	0.05 ± 0	0.04 ± 0	0.04 ± 0	0.05 ± 0	0.05 ± 0	0.05 ± 0
18:0_20:2	0.66 ± 0.04	0.64 ± 0.04	0.64 ± 0.05	0.64 ± 0.04	0.62 ± 0.03	0.62 ± 0.03	0.63 ± 0.03	0.62 ± 0.03	0.62 ± 0.02	0.62 ± 0.02	0.63 ± 0.03	0.61 ± 0.02
16:0_22:2	0.17 ± 0	0.17 ± 0.01	0.16 ± 0	0.17 ± 0	0.18 ± 0	0.19 ± 0	0.18 ± 0.01	0.18 ± 0.01	0.17 ± 0.01	0.18 ± 0	0.18 ± 0	0.18 ± 0.01
16:0_22:4	0.28 ± 0.05	0.3 ± 0.04	0.26 ± 0.04	0.24 ± 0.04	0.24 ± 0.04	0.25 ± 0.04	0.26 ± 0.04	0.25 ± 0.03	0.27 ± 0.04	0.26 ± 0.04	0.27 ± 0.03	0.25 ± 0.04
16:1_22:4	0.05 ± 0.01	0.05 ± 0.01	0.04 ± 0.01	0.04 ± 0.01	0.04 ± 0.01	0.04 ± 0.01	0.04 ± 0.01	0.04 ± 0.01	0.04 ± 0.01	0.05 ± 0.01	0.05 ± 0.01	0.04 ± 0.01
18:0_20:3_ω6	1.36 ± 0.05	1.46 ± 0.04	1.34 ± 0.05	1.3 ± 0.06	1.22 ± 0.06	1.16 ± 0.06	1.27 ± 0.06	1.16 ± 0.07	1.2 ± 0.04	1.29 ± 0.04	1.22 ± 0.08	1.16 ± 0.05
18:0_20:3_ω9	1.02 ± 0.02	0.97 ± 0.01	1.02 ± 0.02	0.89 ± 0.03	0.83 ± 0.02	0.73 ± 0.02	0.87 ± 0.05	0.82 ± 0.01	0.78 ± 0.02	0.93 ± 0.01	0.89 ± 0.04	0.9 ± 0.05
18:0_20:4	2.35 ± 0.35	2.44 ± 0.26	2.32 ± 0.24	1.74 ± 0.05	1.61 ± 0.09	1.36 ± 0.12	1.74 ± 0.04	1.59 ± 0.12	1.63 ± 0.15	1.99 ± 0.26	1.73 ± 0.15	1.61 ± 0.06
18:1_20:3_ω6	0.87 ± 0.01	0.85 ± 0.05	0.84 ± 0.02	0.68 ± 0.03	0.63 ± 0.02	0.57 ± 0.02	0.67 ± 0.03	0.59 ± 0.02	0.6 ± 0.02	0.7 ± 0	0.63 ± 0.01	0.59 ± 0.02
18:1_20:3_ω9	0.44 ± 0.03	0.42 ± 0.02	0.44 ± 0.02	0.38 ± 0.01	0.35 ± 0.01	0.31 ± 0.02	0.36 ± 0.02	0.34 ± 0.03	0.32 ± 0.02	0.39 ± 0.02	0.36 ± 0	0.34 ± 0.01
18:1_20:4	1.88 ± 0.19	1.96 ± 0.21	1.84 ± 0.19	1.3 ± 0.08	1.12 ± 0.06	0.89 ± 0.1	1.3 ± 0.12	1.05 ± 0.09	1.09 ± 0.08	1.44 ± 0.11	1.29 ± 0.04	1.16 ± 0.05
16:0_22:5_ω3	0.59 ± 0.04	0.56 ± 0.04	0.54 ± 0.04	0.43 ± 0.03	0.42 ± 0.02	0.36 ± 0.03	0.46 ± 0.05	0.4 ± 0.03	0.39 ± 0.01	0.49 ± 0.01	0.46 ± 0.02	0.41 ± 0.02
16:0_22:5_ω6	0.05 ± 0	0.04 ± 0	0.04 ± 0.01	0.04 ± 0	0.04 ± 0	0.03 ± 0	0.04 ± 0	0.03 ± 0	0.03 ± 0	0.04 ± 0	0.04 ± 0	0.04 ± 0
18:1_20:5_ω3	0.21 ± 0.02	0.18 ± 0.01	0.2 ± 0.01	0.12 ± 0.01	0.09 ± 0	0.07 ± 0.01	0.12 ± 0.01	0.08 ± 0	0.09 ± 0	0.13 ± 0	0.11 ± 0	0.09 ± 0
18:1_20:5_ω6	0.03 ± 0	0.02 ± 0	0.03 ± 0	0.02 ± 0	0.01 ± 0	0.01 ± 0	0.02 ± 0	0.01 ± 0	0.01 ± 0	0.02 ± 0	0.02 ± 0	0.02 ± 0
16:0_22:6	0.31 ± 0.01	0.27 ± 0.01	0.3 ± 0.01	0.19 ± 0.02	0.16 ± 0.01	0.12 ± 0.01	0.19 ± 0.03	0.13 ± 0.01	0.14 ± 0.01	0.23 ± 0	0.2 ± 0.01	0.15 ± 0.01
16:1_22:5_ω3	0.07 ± 0.01	0.07 ± 0.01	0.06 ± 0.01	0.05 ± 0	0.05 ± 0	0.04 ± 0	0.05 ± 0.01	0.05 ± 0	0.04 ± 0	0.06 ± 0	0.06 ± 0	0.05 ± 0
16:1_22:5_ω6	0.02 ± 0	0.02 ± 0	0.02 ± 0	0.01 ± 0	0.01 ± 0	0.01 ± 0	0.02 ± 0	0.01 ± 0	0.01 ± 0	0.02 ± 0	0.02 ± 0	0.02 ± 0
18:0_22:5_ω3	0.41 ± 0.01	0.46 ± 0	0.45 ± 0	0.35 ± 0.01	0.31 ± 0	0.27 ± 0.01	0.34 ± 0.01	0.3 ± 0.01	0.29 ± 0.01	0.37 ± 0.02	0.32 ± 0	0.3 ± 0.01
18:0_22:5_ω6	0.09 ± 0	0.08 ± 0	0.08 ± 0	0.06 ± 0	0.06 ± 0	0.05 ± 0	0.06 ± 0	0.06 ± 0	0.06 ± 0	0.06 ± 0	0.06 ± 0	0.06 ± 0
18:0_22:4	0.55 ± 0.04	0.56 ± 0.03	0.53 ± 0.03	0.43 ± 0.01	0.41 ± 0.02	0.41 ± 0.03	0.42 ± 0.01	0.41 ± 0.01	0.43 ± 0.02	0.45 ± 0.01	0.41 ± 0	0.4 ± 0.02
18:1_22:4	0.27 ± 0.04	0.28 ± 0.04	0.27 ± 0.04	0.23 ± 0.03	0.22 ± 0.04	0.23 ± 0.03	0.23 ± 0.03	0.22 ± 0.02	0.23 ± 0.02	0.24 ± 0.03	0.23 ± 0.03	0.23 ± 0.03
18:0_22:6	0.37 ± 0.03	0.34 ± 0.02	0.37 ± 0.01	0.24 ± 0.01	0.19 ± 0.01	0.15 ± 0	0.24 ± 0.01	0.17 ± 0	0.18 ± 0	0.26 ± 0.01	0.2 ± 0.01	0.17 ± 0.01
18:1_22:5_ω3	0.42 ± 0.01	0.43 ± 0.02	0.42 ± 0.01	0.3 ± 0.01	0.28 ± 0.01	0.22 ± 0.02	0.3 ± 0.03	0.25 ± 0.01	0.25 ± 0	0.33 ± 0.01	0.29 ± 0	0.25 ± 0
18:1_22:5_ω6	0.09 ± 0	0.08 ± 0	0.08 ± 0	0.07 ± 0	0.06 ± 0	0.05 ± 0	0.06 ± 0.01	0.05 ± 0.01	0.06 ± 0	0.07 ± 0.01	0.07 ± 0	0.06 ± 0
18:1_22:6	0.47 ± 0.03	0.46 ± 0.02	0.48 ± 0.02	0.3 ± 0.03	0.24 ± 0.02	0.2 ± 0.01	0.3 ± 0.04	0.21 ± 0.02	0.22 ± 0.02	0.33 ± 0.02	0.27 ± 0.02	0.24 ± 0.03
18:2_22:5_ω3	0.03 ± 0	0.03 ± 0	0.03 ± 0	0.02 ± 0	0.01 ± 0	0.01 ± 0	0.02 ± 0	0.02 ± 0	0.01 ± 0	0.02 ± 0	0.02 ± 0	0.01 ± 0
18:2_22:5_ω6	0.01 ± 0	0.01 ± 0	0.01 ± 0	0.01 ± 0	0.01 ± 0	0.01 ± 0	0.01 ± 0	0.01 ± 0	0.01 ± 0	0.01 ± 0	0.01 ± 0	0.01 ± 0

Supplementary Fig. 10. Short-term effect of SCs on the PC fatty acid composition of triple-negative breast cancer cells. MDA-MB-231 cells ($3.12\times10^{6}/75\ cm^{2}$) were treated with vehicle (‘w/o’, 0.1% DMSO), RSL3 (1 µM), RSL3 plus Fer-1 (3 µM), **1**, **2**, or **3** (1–10 µM), and non-oxidized PC species were analyzed by UPLC-MS/MS. Numbers indicate the proportion of individual PC species relative to total PC (100%) and the color expresses percentage changes relative to vehicle. Data are expressed as mean ± SEM from n = 3 independent experiments.

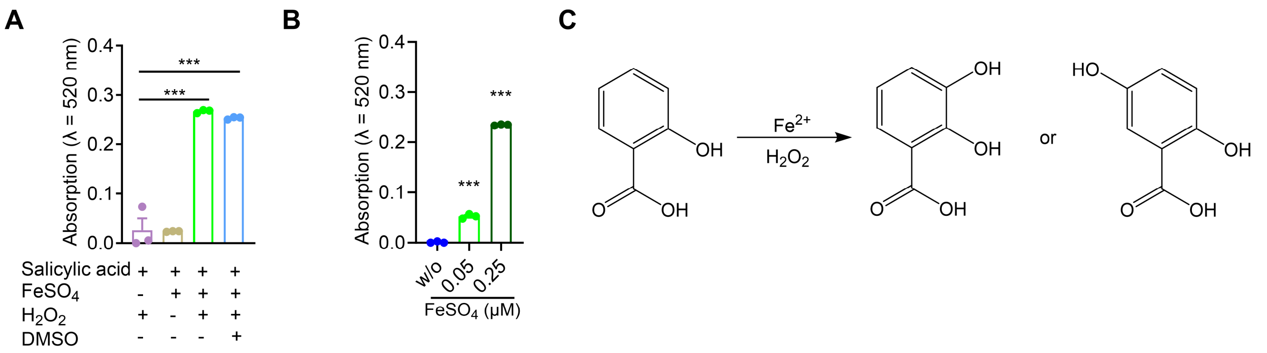

Supplementary Fig. 11. Fenton-active Fe(II) efficiently hydroxylates salicylic acid in the presence of H_2O_2. A, B. Extent of OH-radical-dependent oxidation of salicylic acid (0.52 mM) by vehicle ('w/o', H_2O), $FeSO_4$ (A: 0.25 mM; B: as indicated), or $FeSO_4$ plus DMSO (2.3%) in the presence or absence of H_2O_2 (225 mM) within 30 min at room temperature. Data are expressed as mean ± SEM from n = 3 independent experiments. $^{***}P < 0.001$ compared to vehicle control; ordinary one-way ANOVA + Dunnett's *post hoc* tests. C. Scheme illustrating the Fenton reaction-mediated hydroxylation of salicylic acid by Fe(II) and H_2O_2, involving the generation of OH-radicals.

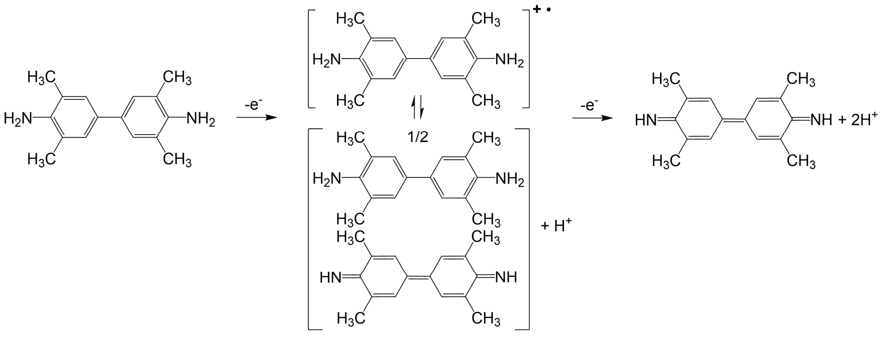

Supplementary Fig. 12. Scheme illustrating the oxidation of TMB to colored products by one- and two-electron transfer reactions.

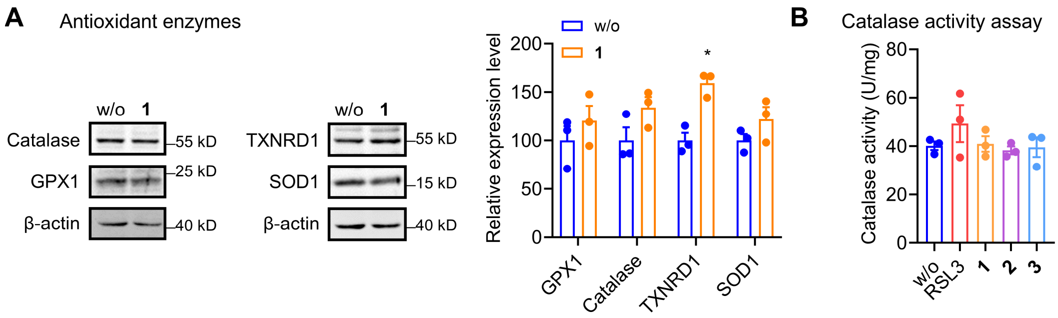

Supplementary Fig. 13. Compound 1 induces a moderate antioxidant response. MDA-MB-231 cells (5×10^5/well of a 6-well plate) were incubated (after 24 h) with vehicle ('w/o', 0.1% DMSO), RSL3 (1 µM), **1**, **2**, or **3** (0.3 µM) for another 24 h. A. Protein expression of antioxidant enzymes. B. Cellular catalase activity. Data are expressed as mean ± SEM from n = 3 independent experiments. $^{*}P < 0.05$ compared to vehicle control; repeated measures two-way ANOVA + Sidak's tests (A) or repeated measures one-way + Dunnett's tests (B).

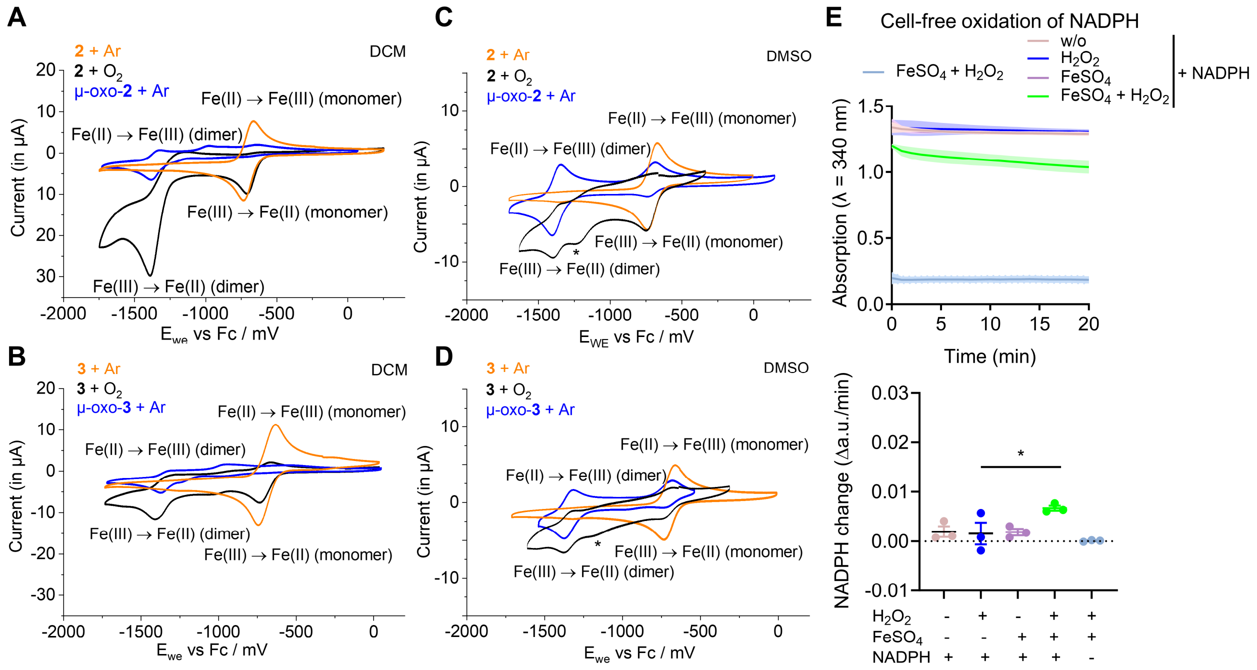

Supplementary Fig. 14. SCs contain redox-active iron and undergo reversible dimerization depending on the redox state. A-D. Cyclic voltammograms of monomeric and dimeric SCs (1 mM) under oxygen and inert gas (argon) atmosphere. Compounds **2** and μ-oxo-**2** (A, B) or **3** and μ-oxo-**3** (B, D) were dissolved in DCM (A, B) or DMSO (C, D). The asterisk (*) indicates oxygen reduction. E. Photometric analysis of the oxidation of NADPH (0.5 mM) by H_2O_2 (37 mM) and vehicle (‘w/o’, 1% DMSO) or $FeSO_4$ (0.1 mM) or in the absence of H_2O_2. The median and single value plot indicates the oxidation rate (change in absorption per min). Data are representative of n ≥ 5 scans (A-D) or expressed as mean ± SEM from n = 3 (E) independent experiments. $^{*}P < 0.05$ compared to vehicle control; ordinary one-way ANOVA + Dunnett’s *post hoc* tests (E).
